# Gene Expression Changes in the Prefrontal Cortex, Anterior Cingulate Cortex and Nucleus Accumbens of Mood Disorders Subjects That Committed Suicide

**DOI:** 10.1371/journal.pone.0035367

**Published:** 2012-04-30

**Authors:** Adolfo Sequeira, Ling Morgan, David M. Walsh, Preston M. Cartagena, Prabhakara Choudary, Jun Li, Alan F. Schatzberg, Stanley J. Watson, Huda Akil, Richard M. Myers, Edward G. Jones, William E. Bunney, Marquis P. Vawter

**Affiliations:** 1 Department of Psychiatry and Human Behavior, University of California Irvine, Irvine, California, United States of America; 2 Center for Neuroscience, University of California Davis, Davis, California, United States of America; 3 Department of Human Genetics, University of Michigan, Ann Arbor, Michigan, United States of America; 4 Department of Psychiatry and Behavioral Sciences, Stanford University, Palo Alto, California, United States of America; 5 Molecular and Behavioral Neuroscience Institute, University of Michigan, Ann Arbor, Michigan, United States of America; 6 HudsonAlpha Institute for Biotechnology, Huntsville, Alabama, United States of America; The University of Melbourne, Australia

## Abstract

Suicidal behaviors are frequent in mood disorders patients but only a subset of them ever complete suicide. Understanding predisposing factors for suicidal behaviors in high risk populations is of major importance for the prevention and treatment of suicidal behaviors. The objective of this project was to investigate gene expression changes associated with suicide in brains of mood disorder patients by microarrays (Affymetrix HG-U133 Plus2.0) in the dorsolateral prefrontal cortex (DLPFC: 6 Non-suicides, 15 suicides), the anterior cingulate cortex (ACC: 6NS, 9S) and the nucleus accumbens (NAcc: 8NS, 13S). ANCOVA was used to control for age, gender, pH and RNA degradation, with *P*≤0.01 and fold change±1.25 as criteria for significance. Pathway analysis revealed serotonergic signaling alterations in the DLPFC and glucocorticoid signaling alterations in the ACC and NAcc. The gene with the lowest p-value in the DLPFC was the 5-HT2A gene, previously associated both with suicide and mood disorders. In the ACC 6 metallothionein genes were down-regulated in suicide (MT1E, MT1F, MT1G, MT1H, MT1X, MT2A) and three were down-regulated in the NAcc (MT1F, MT1G, MT1H). Differential expression of selected genes was confirmed by qPCR, we confirmed the 5-HT2A alterations and the global down-regulation of members of the metallothionein subfamilies MT 1 and 2 in suicide completers. MTs 1 and 2 are neuro-protective following stress and glucocorticoid stimulations, suggesting that in suicide victims neuroprotective response to stress and cortisol may be diminished. Our results thus suggest that suicide-specific expression changes in mood disorders involve both glucocorticoids regulated metallothioneins and serotonergic signaling in different regions of the brain.

## Introduction

Suicide is one of the leading causes of death in the world particularly for young males and projections of global mortality indicate that suicide rates are rising [1] and will remain a leading cause of injury and death worldwide [2]. The number of suicides is approximately 1 million per year in the world [3]. In the United States suicide is the eleventh leading cause of death for all ages and the third leading cause among adolescents [4]. One third of those suicides visited a health care professional within a week before committing suicide [4]. This suggests that better methods are needed in assessment of suicidal intent in at risk populations.

Mood disorders (major depressive disorder (MDD) and bipolar disorder (BD)) constitute prominent risk factors for suicide [5]–[7]. Accordingly, lifetime risk for suicide in mood disorders patients is particularly high when compared to the general population [8]–[12]. In BD patients suicide attempts are specifically associated with depressive episodes rather than manic episodes [13]. Despite current advances in the understanding of suicide predisposing factors, existing diagnostic algorithms are inadequate and the identification of individuals at high risk for suicide is frequently missed [14].

Suicide and suicidal behaviors have been consistently linked to monoaminergic (serotonin, norepinephrine and dopamine) neurotransmission alterations [15]–[17] and to alterations in the hypothalamic-pituitary-adrenal (HPA) axis [18]–[20]. Postmortem receptor/transporter binding studies have also confirmed alterations in the number and affinity of monoaminergic receptors in the brains of suicides [17], [21]. Interestingly, both serotonergic neurotransmission imbalance and HPA axis over-activity are related to stressful events and were proposed as risk factors for suicide [22]. Recent evidence has shown that childhood abuse alters HPA stress responses and increases the risk of suicide through epigenetic regulation of glucocorticoid receptor expression underlining the role played by the HPA-stress response in the susceptibility to suicide [23].

Recently, microarray technology has been used to investigate in post-mortem brain global gene expression alterations associated with mood disorders and suicide compared to psychiatrically normal controls [24]. A number of studies specifically explored gene expression alterations in post-mortem brains of suicides versus control subjects in prefrontal regions [25]–[30] and in the limbic system [31], [32] with very few replicated results due probably to methodological and demographic differences in the samples. These studies focused mainly on the investigation of genes differentially expressed between psychiatric cases that committed suicide and controls, but did not compare the psychiatric patients who commit suicide from those who do not. To our knowledge, only two recent articles investigated suicide specific changes in the brain by comparing suicide versus non-suicide psychiatric patients. Garbett et al. observed a prefrontal cortical down-regulation of the HTR2A receptor in subjects with schizophrenia who committed suicide using Affymetrix arrays [33]. Also, Kim et al. (2007) reanalyzed a large microarray data set to investigate gene expression in major depression and schizophrenic patients who did and did not commit suicide, in the prefrontal cortex (BA10/46) [30]. They found alterations on 2 genes across diagnoses, PLSCR4 (phospholipid scramblase 4) and EMX2 (empty spiracles homolog 2 (Drosophila)) and several members of the metallothionein subfamily 1 (MT1E, MT1X, MT1M, MT1G, MT1H, MT1F) as being specifically down-regulated among the schizophrenics who committed suicide [30].

Mental disorders and particularly mood disorders contribute significantly but not exclusively to the risk of suicide. Determining the expression changes in the brain that differentiate suicide from non-suicide mood disorder brains should help identify the genes specifically involved in suicide. In this study, we investigated a cohort of mood disorder subjects (BD and MDD) for gene expression changes specifically associated with suicide in the DLPFC, ACC, and NAcc, three regions commonly associated with suicidal behavior alterations, mood, control of impulses, motivation, reward and pleasure, behaviors know to be altered in mood disorders.

## Methods

### Subjects

Brain samples were obtained at the University of California, Irvine/Davis Brain Repository through a uniform process approved by the Institutional Review Board. Written informed consent was obtained from the next-of-kin of the deceased. All subjects went through an extensive review of multiple sources of information including the medical examiner’s conclusions, coroner’s investigation, medical and psychiatric records, toxicology results, and interviews of the decedents’ next-of-kin, in order to rule out as much as possible any neurological disorder. Additionally, a neuropathological examination of each brain was conducted in order to exclude cases with cerebrovascular disease (infarcts or hemorrhages), subdural hematoma, or other significant pathological features. Our psychological autopsy protocol is based largely on procedures validated by Kelly and Mann [34]. Details of death are evaluated in order to assign an Agonal Factor Score to each subject in accordance with previous guidelines hardy [35], [36]. Previous work consistently showed an association between agonal factors and tissue quality [37]–[40]. Brains are initially screened for agonal factors at the time of consent in order to exclude brains with factors potentially affecting nucleic acids and proteins. Subjects with history of drugs or alcohol dependence were also excluded from the present sample. Non-suicide subjects, including mood disorder subjects and normal controls, died rapidly from either cardiac related events or motor-vehicle accidents.

The human brain dissection and freezing protocol is described in detail elsewhere [41]. Briefly, the whole brain is first inspected and photographed before the brainstem and cerebellum are separated from the cerebrum. The cerebrum is coronally sliced from anterior to posterior and slices are photographed, labeled, and bagged before being frozen. Small 5 cm squares are dissected for both pH and RNA quality measurements. Freezing is done by placing the bagged slices between super-cooled aluminum plates, then in a pre-cooled freezing rack before putting the whole rack in a −140°C freezer for a minimum of 10 minutes. The brains are stored in −80°C freezers until dissected and processed for extractions. The brain regions were dissected on dry ice from the left hemisphere of frozen tissue according to visible landmarks near the regions of interest under the direction of Dr E. G. Jones. Grey matter punches were taken from the cortical regions and from the middle of NAcc region.

Grey matter brain samples (25 mg) were used to extract total RNA with the Trizol method (Invitrogen, Carlsbad, CA), followed by a cleaning up using a silica based mini-spin column (Qiagen RNeasy Mini Kit, Valencia, CA). RNA integrity was evaluated using 18S and 28S ratios and RNA integrity numbers (RIN) using the 2100 Agilent Bioanalyzer (Santa Clara, CA). All subjects were clinically characterized with the psychological autopsy method and died suddenly without prolonged agonal state.

### Microarray Analysis

Cel files from Affymetrix HG-U133 Plus2.0 chips were processed using the RMA algorithm. Statistical analysis was performed using Partek (St. Louis, MO). An ANOVA model was used to identify the differentially expressed genes between the non suicides and the suicides in a sample of mood disorder patients controlling for age, gender, RIN, and pH. Additionally, the AffyRNAdeg function was used to generate 3′/5′ slopes indicating RNA degradation and used as covariate in the ANOVA model [42]. Genes were considered as differentially expressed if they had a *P*-value of less than 0.01 and a fold change of ±1.25. Pathway analysis was performed using Ingenuity (Redwood City, CA) and the differentially expressed probe sets in each region.

Outlier detection was conducted in order to exclude problematic arrays using a combination of principal component analysis (PCA), and RNAdeg Slopes. In the DLPFC, 21 subjects were analyzed by microarrays, 15 suicides (3 females/12 males) and 6 non-suicides (2 females/4 males). In the ACC, a total of 15 subjects were included in the microarray analysis, 9 suicides (3 females/6 males) and 6 non-suicides (3 females/3 males). In the NAcc a total of 21 subjects were analyzed by microarrays, 13 suicides (3 females/10 males) and 8 non-suicides (2 females/6 males). For the microarray experiment sample (cohort 1), demographic and physiological variables used for the ANOVA model and to assess the RNA quality (age, pH, PMI or RIN numbers) can be found in **Table 1** for the group averages and standard deviation and **Table S1** for the detailed information. The entire dataset has been deposited to the Gene Expression Omnibus (GEO) with the Accession Number GSE6306 (**Table S6**).

**Table 1:** Demographic and physiological details for the subjects included in the microarray gene expression study sample (cohort 1).

	DLPFC		ACC		NAcc	
	Suicide (n = 15)	NS (n = 6)	Suicide (n = 9)	NS (n = 6)	Suicide (n = 13)	NS (n = 8)
**Age (Mean±SD)**	44±14	42±17	43±9	53±10	43±9	54±8
**pH (Mean±SD)**	6.8±0.3	6.9±0.4	6.9±0.3	6.7±0.3	6.9±0.3	6.8±0.2
**PMI (Mean±SD)**	25±4.2	23±8.3	24±5.0	25±5.0	24±4.7	25±6.6
**Slope (Mean±SD)**	3.3±0.6	2.9±0.3	3.6±0.3	4.0±0.5	3.0±0.2	2.9±0.4
**RIN (Mean±SD)**	7.9±0.5	7.9±0.6	8.0±0.4	8.0±0.3	8.1±0.4	8.0±0.5

Mean±standard deviation values (SD) are presented. The slope was calculated using the AffyRNAdeg function. DLPFC: dorsolateral prefrontal cortex, ACC: anterior cingulate cortex, NAcc: nucleus accumbens.

### Confirmation of Differential Expression

Confirmation of a subset of genes was done was done in an extended cohort including also normal controls by quantitative real-time polymerase chain reaction (qPCR) using the SYBR Green chemistry. Normalization of the qPCR results was performed using the geNorm method developed by Vandesompele et al. [43], this method consists on a factor calculation normalization based on the geometric mean of multiple endogenous control genes, in this case we used CAMK2A and SDHA. Samples were run in triplicate and confirmation of the results was determined by ANOVA controlling for age, pH, and diagnosis. Primers were designed to amplify regions close to the corresponding target sequences in the microarray experiments and spanning two exons in order to only amplify mRNA (primers are available on request). Experiments were performed in a 384 well plate in a total volume of 10 µl on a 7900 HT real time sequence detector (Applied Biosystems, Foster City, CA). The qPCR confirmation cohort (cohort 2) also included normal controls and showed no differences in terms of age, pH, gender or PMI between the groups (**Table 2** and **Table S2**).

**Table 2:** Demographic and physiological details for the subjects included in the qPCR validation sample (cohort 2).

	DLPFC			ACC			NAcc		
	Suicide (n = 20)	NS (n = 11)	Control (n = 27)	Suicide (n = 10)	NS (n = 7)	Controls (n = 18)	Suicide (n = 13)	NS (n = 7)	Control (n = 10)
**Age (Mean±SD)**	44±12	52±11	50±15	43±11	54±9	49±14	46±9	49±9	41±11
**pH (Mean±SD)**	6.9±0.2	6.9±0.2	6.9±0.3	7.0±0.2	6.9±0.2	7.0±0.2	6.9±0.2	6.9±0.2	6.9±0.2
**PMI (Mean± SD)**	26±4	24±6	20±7	24±5	26±5	20±5	25±5	24±6	21±5
**RIN (Mean ± SD)**	7.9±0.5	7.8±0.7	7.8±0.6	8.2±1	8.3±1	8.0±1	NA	NA	NA

Mean±standard deviation values (SD) are presented. DLPFC: dorsolateral prefrontal cortex, ACC: anterior cingulate cortex, NAcc: nucleus accumbens, NA: not available.

## Results

No significant differences between the suicide and non-suicide groups were observed for the major demographic variables such as pH, age, RIN, PMI and RNAdeg slope (**Table 1** and **Table S1**). In order to control for possible confounding effects, demographic variables, such as diagnosis and age, and RNA quality measures (RIN and slope) were used as covariates.

**Figure 1:**
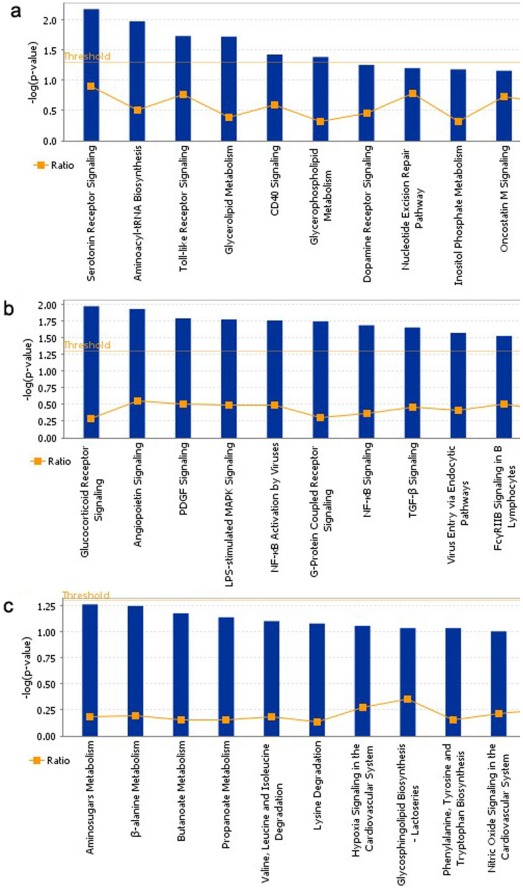
Canonical pathway enrichment analysis using Ingenuity. **A** In the DLPFC. **B** In the ACC. **C** In the NAcc.

A total of 161 genes were found to be differentially expressed in the DLPFC (**Table S3**). The most statistically significant gene in this region was the 5-HT2A gene (P = 0.00022, FC = −1.28), previously identified as being implicated in both mood disorders and in suicide consistently. The decrease of 5-HT2A expression in the suicide group was confirmed by qPCR (data not shown). Enrichment of canonical pathways analysis using Ingenuity confirmed that the serotonin receptor signaling pathway was altered in suicide brain tissue (**Fig. 1** ***a***). Another gene of interest previously shown to be involved in suicide [44] and that was altered in the DLPFC was the adenylate cyclase 1 (brain) (ADCY1) gene that codes for a brain specific form of adenylate cyclase responsible of the production of the second messenger cyclic AMP, ADCY was significantly down-regulated in suicide versus non-suicide patients. Finally, period homolog 1 (PER1), a major component of the circadian rhythm system, was also down-regulated in suicides compared to non-suicides. Clock genes have been in the past associated with mood disorders [45] but not specifically with suicide. In summary, the DLPFC data shows it is possible to detect significant expression alterations in human brain areas between suicides and non-suicides brains and confirms the involvement of serotonergic and second messenger system alterations in suicide.

**Figure 2:**
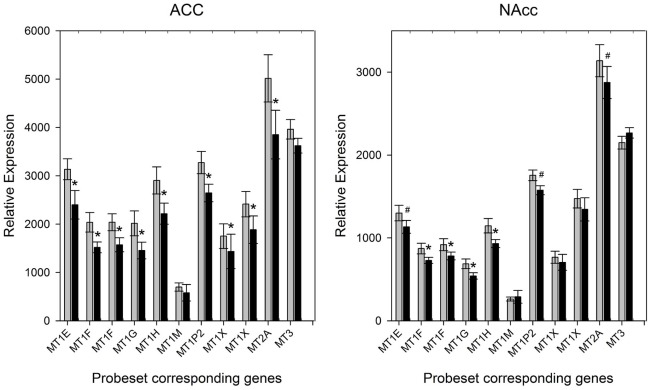
Expression levels of 11 metallothionein probe sets corresponding to 9 gene as measured by microarrays (HG-U133Plus 2.0) in the ACC and NAcc of non-suicide (grey bars) and suicide (black bars) mood disorder subjects. Significant genes/probe sets are labeled by * (P<0.01) and # (P<0.05) signs.

In the ACC, 139 annotated probe sets were differentially expressed between the suicides and non-suicides cases (**Table S4**). Of these, eight probe sets corresponded to seven metallothionein family members (MT1E, MT1M, MT1F, MT1G, MT1H, MT1X, MT2A), located as a cluster on chromosome 16q13 (Table 3). All these probe sets were significantly down-regulated in suicide victims with fold changes varying from −1.46 to −1.83. Pathway analysis conducted using Ingenuity on the 139 differentially expressed probe sets revealed that the most over represented pathway was glucocorticoid receptor signaling (**Fig. 1** ***b***). Further exploration of the probe sets altered in the glucocorticoids receptor signaling pathway revealed a global down-regulation of metallothionein subfamilies 1 and 2 genes. In fact, a closer look at the expression levels of all the metallothionein genes revealed that most of the genes in subfamilies 1 and 2 where down-regulated in the ACC, including pseudogenes co-localized in 16q13 (**Fig. 2**). Interestingly, the brain specific MT3 gene was highly expressed, but showed no differences in gene expression like subfamilies 1 and 2.

**Table 3:** qPCR Confirmation of decreased expression of metallothionein subfamilies 1 and 2.

	NACC		ACC	
	*P*-value	T	*P*-value	T
MT1E	**0.09**	3.25	**0.02**	6.36
MT1F	**0.02**	7.23	**0.05**	4.14
MT1G	**0.01**	7.63	**0.01**	7.67
MT1H	**0.01**	8.96	**0.00**	14.19
MT1X	**0.03**	5.65	**0.02**	5.99
MT2A	**0.03**	5.83	**0.04**	4.72

Significant differences are in bold for one tailed t-test confirming the direction of the change.

In the NAcc, 146 probe sets were differentially expressed between suicides and non-suicides (**Table S5**). Three metallothionein genes were also down-regulated in the NACC (MT1F, MT1G, MT1H) among the suicide victims and like in the ACC several others were also down-regulated although at a *P*<0.05 level. IPA did not reveal any canonical pathway as being over-represented, as opposed to the ACC (**Fig. 1** **** ***c***), only three MT genes passed the significance criteria. However, 7 probe sets in total were differentially expressed at the 0.05 level confirming the overall dysregulation in this family of genes (**Fig. 2**). The Wolfram syndrome gene (WFS1), previously associated with psychiatric disorders and suicide [46]–[48], was also down-regulated among the suicide victims in the NACC.

### Confirmation of Results by qPCR

**Figure 3:**
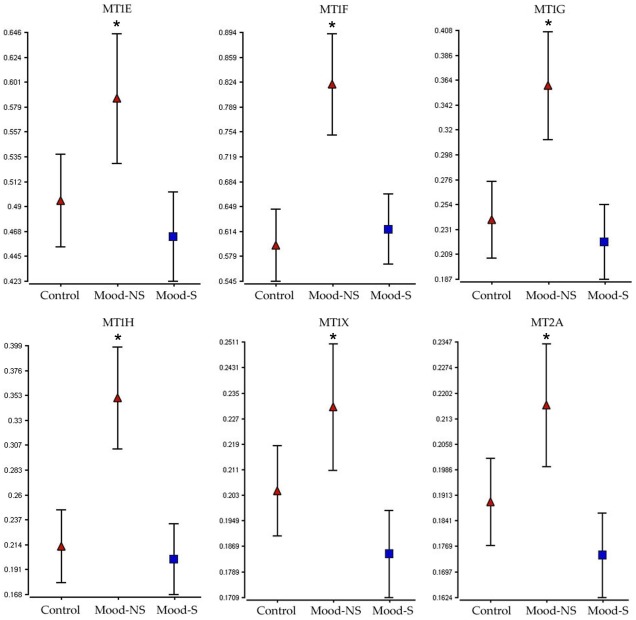
Expression levels in the NACC of six metallothionein genes in as measured by qPCR in psychiatrically normal controls and mood disorder patients that did (Mood-S) or did not commit suicide (Mood-NS). * denotes significance at the P<0.05 versus the Mood-S group.

Group averages for demographic and physiological variables for the subjects (cohort 2) included in the confirmation experiments per region can be seen in Table 2 and for subjects in cohort 2 used in this analysis can be seen in Table 2 and detailed demographic data in table S2. All the subjects used the validation cohort had high pH (mean = 6.9) and RIN values (mean = 8), and an average the PMI of 23 hours (8.5 to 37 hours). Metallotionein gene expression down-regulation in both the ACC and NACC was confirmed by qPCR for all the functional variants in sub-families 1 and 2 known to be expressed in the brain (MT1E, MT1F, MT1G, MT1H, MT1X, MT2A). In summary, the pattern of metallothionein expression was confirmed to be lower in the suicides compared to the non-suicides in both the ACC and NACC for all the genes examined (**Figs. 3** **and** **4**). When compared to normal controls, metallothionein levels tended to be elevated in the non-suicide mood disorder group, especially in the NAcc, while lower specifically in the suicide group.

**Figure 4:**
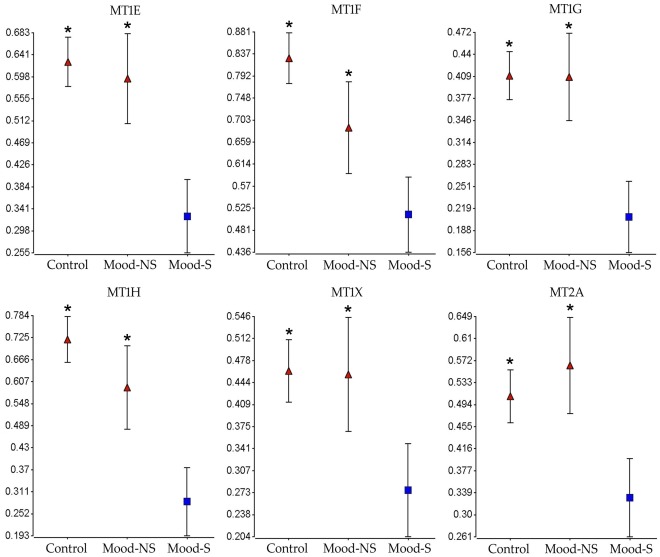
qPCR confirmation in the ACC. Expression levels in the ACC of six metallothionein genes measured by qPCR and the GE-Norm method between psychiatrically normal controls and mood disorder patients that did (Mood-S) or did not commit suicide (Mood-NS). * denotes significance at the P<0.05 versus the Mood-S group.

## Discussion

A gene expression survey by microarrays in three brain regions (DLPFC, NAcc and ACC) revealed alterations in the serotonergic and second messenger systems in the DLPFC and a consistent down-regulation of several members of the metallothionein subfamilies 1 and 2 genes in the NAcc and ACC in mood disorder suicides. Our strategy consisted on using high quality brains (short PMI, high pH, high RIN) with no history of drug dependence, as well as the development of a general linear model in which factors known to influence RNA quality and expression were taken into account when exploring the suicide specific expression changes. This is to our knowledge the first study investigating suicide specific changes in mood disorders in three highly relevant areas as they are involved in the control of impulses and in mood disorders (DLPFC, ACC) and in motivation-reward mechanisms (NAcc).

We studied gene expression changes in the DLPFC, an area previously associated with both mood disorders, control of impulses and suicide. Interestingly, the most statistically significant gene differentially expressed in suicide victims was the 5-HT2A serotonergic receptor, with a1.28 fold-change reduction in mood disorder subjects that committed suicide versus non-suicide mood disorder subjects. Genetic association studies have often shown that mutations in the 5-HT2A receptor are associated with mood disorders and suicide [49], as well as with the response to antidepressants [50]. Additionally, gene expression post-mortem studies have revealed alterations 5-HT2Areceptor levels in mood disorder subject suicides when compared to controls. However the direction of the alterations in 5-HT2A is controversial, with some reports showing elevations in the 5-HT2A binding and expression [51]–[55] while others showed reduced levels or no differences [56]–[58]. Recently, a well designed study including matched suicides and controls also confirmed the absence of changes in 5-HT2 binding but reported a decrease in neuronal densities in the prefrontal cortex in a sample of predominantly major depressed suicides [59]. In another recent study designed to investigate 5-HT2A levels in major depression, Shelton et al. reported an increase in 5-HT2A expression in major depression subjects, but reported no differences when comparing suicides versus non-suicide subjects [55]. In this study, we aimed to investigate suicide specific changes in mood disorder patients and observed a significant decrease in 5-HT2A levels in suicide which can at least partially explain previous discordant findings when comparing mood disorder to controls or suicides to controls. The reduction in 5-HT2A levels is also in concordance with another study comparing suicide versus non-suicide schizophrenia subjects using also Affymetrix microarrays [33], suggesting that this reduction in 5-HT2A levels might be involved in suicide irrespective of the diagnosis. We also observed decreased expression in suicide group of the brain specific ADCY1, a brain specific form of adenylate cyclase, suggesting altered neurotransmission and cyclic AMP signaling in suicide and confirms previous observations pointing to cAMP signaling alterations in suicide and mood disorders [60]–[63] and in animal models of depression [64]. Both serotonergic alterations and second messenger systems underline global neurotransmission alterations in the DLPFC that seem to play a role in the predisposition to commit suicide.

In both the ACC and NAcc decreased expression was observed in gene members of two subfamilies (1 and 2) of the metallothionein super family in the suicides versus the non-suicides mood disorder subjects. These changes were confirmed successfully Metallothioneins are small single chain proteins with a high content of cysteine residues organized in specific sequences enabling the formation of thiolate cluster and the binding of certain metals react to oxidative stress, glucocorticoids and inflammatory mediators [65]. There are four subfamilies of metallothioneins proteins in mammals corresponding to types 1 to 4, all metallothionein genes are closely located in chromosome 16q13. MT1 and MT2 are expressed ubiquitously and highly co-expressed, whereas MT4 is not expressed in the brain and MT3 is brain specific and expressed mainly in hippocampus amygdala and cortex in zinc enriched neurons and astrocytes [66]. Consistent with this, MT3 was also observed to be expressed in the three brain areas investigated but was not differentially expressed like MT1 and 2 subfamilies. MT3, unlike MT 1 and 2 is not regulated by glucocorticoids and stress, which can explain this difference [67]–[72].

MT1 and MT2 are not only known to be responsive to several forms of stress and to glucocorticoids [73], they also play an important role in neuroprotection [66]. In animal models of stress, both MT1 and MT2 mRNA were elevated as much as 30-fold following just 12 h (one cycle) of restraint stress whereas MT3 gene expression was not altered by stress [74]. The induction occurred at the transcriptional level and was mediated by the activation of glucocorticoid receptor [74]. In a stress mouse model, treatment with a glucocorticoid receptor antagonist (RU 486) prior to restraint stress inhibited MT induction by at least 50% indication that at least in part MT induction is under direct control of the glucocorticoid receptor at the transcriptional levels [75]. Glucocorticoids induction of metallothioneins expression is done through the direct binding of the ligand activated glucocorticoid receptor to the glucocorticoid response elements (GRE) in the promoter of MTs [76], [77]. Dexamethasone has been shown for instance to increase significantly the levels of MT and immobilization stress has also been shown to increase selectively MT1 and MT2 of rat brains [70]. MT1 and 2 play a neuroprotective role against stress and glucocorticoids elevation by a mechanism of induced expression of metallothioneins in astrocytes followed by an extracellular release and internalization by neurons after binding to the receptor megalin that mediates the transport of MTs into neurons [78]. Thus, MT1 and 2 sub-families, that play an important role in neuroprotection from the effects of behavioral stress and in response to cortisol, are significantly reduced in mood disorder suicides compared to non-suicides. Interestingly, in another recent microarray study using Affymetrix Exon 1.0 ST arrays, Shelton et al. observed and confirmed by qPCR a decreased expression of MT1M in the BA10 of depressed subjects compared to controls [79].

Alterations in the hypothalamic-pituitary-adrenal (HPA) axis in depressive states and suicide have been consistently observed for more than 40 years [19], [80]. It has also been extensively shown that depressed states are specifically linked to both chronic stress and elevated cortisol levels relevant of HPA axis hyperactivity in depression [81]. Many studies have observed higher baseline cortisol values in young depressed patients [82] and cortisol hypersecretion in depression [83]. We propose in this study that decreased levels of MT1 and 2 can result in a higher sensitivity in suicide completers to the effects of depression-associated cortisol levels in the brain. If these results are confirmed in a larger independent cohort, pharmacological agents that increase the expression of MT 1 and 2 metallothionein sub-families or their transport to neurons by the megalin receptor without necessarily altering HPA axis activity can be envisioned for the treatment and prevention of suicide and suicidal behaviors in mood disorder patients.

The effect of known confounding variables in post-mortem brain gene expression studies (pH, PMI, age, gender, diagnosis and RNA quality) was controlled by conducting an ANCOVA using significant variable as covariates. One of the weaknesses of this study is the relatively small size of the microarray sample. However, we were able to confirm the observed results in a larger sample and to explore MT expression in control subjects also by qPCR in a larger sample in both the ACC and NAcc (**Figs. 3** **and** **4**). Another problem observed was the difference in age between the non-suicides and the suicides in the NAcc and ACC. This was expected as suicide is particularly prevalent in younger populations [2], [2], [7,84] with about 200,000 young people that die every year in the world by suicide [85]. For that reason, we included age into the statistical model in order to exclude genes altered by age only. Ultimately however, MT subfamilies 1 and 2 down-regulation in suicide mood disorder subjects should be tested in peripheral tissue such as blood lymphocytes, where they have been shown to be expressed [86], in order to confirm these results in a clinical population and to investigate the validity of these changes as a biomarker for suicide.

In conclusion, while expression profiling confirmed and further extended the implication of serotonergic genes in suicide in the DLPFC, the present study also reveals a significant decrease in expression of metallothioneins subfamilies1 and 2, a family of genes involved in neuroprotection and in cortisol/stress response, in suicide victims compared to non-suicide mood disorder patients. Because cortisol levels are known to be elevated in depressive states, the observed decreased in MT expression in the suicide group might point to a failed neuroprotective mechanism against cortisol in this group and point to a new marker and possible pharmacological target for suicide and suicidal behavior.

## Supporting Information

Table S1 **Complete demographic variables for the subjects included in the microarray analysis per brain region.** (RIN: Agilent 2100 RNA Integrity Number; MDD: major depressive disorder; BD: bipolar disorder; NS: non suicide; MOD: method of death).
(DOC)

Table S2 **Complete demographic variables for the subjects included in the confirmation qPCR analysis of the DLPFC, ACC and Nacc.** (MDD: major depressive disorder; BD: bipolar disorder; NS: non suicide; PMI: post-mortem interval; MOD: method of death).
(DOC)

Table S3 **Differentially expressed probe sets between suicide and non-suicide mood disorder subjects in the DLPFC.**
(DOC)

Table S4 **Differentially expressed probe sets between suicide and non-suicide mood disorder subjects in the ACC (139).**
(DOC)

Table S5 **Differentially expressed probe sets between suicide and non-suicide mood disorder subjects in the NAcc (146).**
(DOC)

Table S6 **Accession numbers for the chips included in the study.**
(DOC)
