# Supplementary material for: Gene Expression Changes in the Prefrontal Cortex, Anterior Cingulate Cortex and Nucleus Accumbens of Mood Disorders Subjects That Committed Suicide
Source: PLoS One. 2012 Apr 30;7(4):e35367. doi: 10.1371/journal.pone.0035367 (PMC3340369; doi:10.1371/journal.pone.0035367)
Supplement: Table S1 — Complete demographic variables for the subjects included in the microarray analysis per brain region. (RIN: Agilent 2100 RNA Integrity Number; MDD: major depressive disorder; BD: bipolar disorder; NS: non suicide; MOD: method of death). (DOC) [file pone.0035367.s001.doc]

**Supporting table 1.** Complete demographic variables for the subjects included in the microarray analysis per brain region.

| Diagnosis | Gender | Status | RIN | Slope | pH | Age | PMI | Region | MOD |
| --- | --- | --- | --- | --- | --- | --- | --- | --- | --- |
| MD | F | NS | 7.50 | 3.82 | 7.06 | 44 | 25.00 | ACC | Sudden medical condition |
| BP | F | NS | 8.20 | 3.73 | 6.55 | 59 | 27.00 | ACC | Sudden medical condition |
| BP | M | NS | 8.10 | 3.70 | 6.96 | 70 | 27.50 | ACC | Accident |
| MD | M | NS | 8.10 | 3.88 | 6.84 | 50 | 30.80 | ACC | Sudden medical condition |
| MD | M | NS | 8.20 | 3.93 | 6.41 | 48 | 25.50 | ACC | Sudden medical condition |
| BP | F | NS | 7.90 | 5.04 | 6.74 | 49 | 15.90 | ACC | Accident |
| BP | F | Suicide | 7.90 | 4.00 | 7.00 | 36 | 25.50 | ACC | Suicide |
| MD | F | Suicide | 8.00 | 3.59 | 7.16 | 53 | 19.50 | ACC | Suicide |
| MD | M | Suicide | 7.80 | 3.71 | 6.96 | 56 | 13.00 | ACC | Suicide |
| MD | M | Suicide | 8.30 | 3.84 | 7.13 | 34 | 27.50 | ACC | Suicide |
| MD | F | Suicide | 8.40 | 4.12 | 6.76 | 46 | 28.00 | ACC | Suicide |
| MD | M | Suicide | 8.10 | 3.57 | 6.58 | 52 | 23.00 | ACC | Suicide |
| MD | M | Suicide | 7.20 | 3.52 | 6.99 | 40 | 24.00 | ACC | Suicide |
| MD | M | Suicide | 8.10 | 3.62 | 7.18 | 39 | 29.00 | ACC | Suicide |
| MD | M | Suicide | 8.50 | 3.33 | 6.51 | 34 | 22.20 | ACC | Suicide |
| BP | F | NS | 8.20 | 2.59 | 6.55 | 36 | 27.00 | DLPFC | Sudden medical condition |
| BP | M | NS | 7.20 | 2.63 | 6.96 | 19 | 27.50 | DLPFC | Accident |
| MD | F | NS | 8.40 | 2.86 | 7.39 | 28 | 7.60 | DLPFC | Sudden medical condition |
| MD | M | NS | 7.90 | 3.17 | 6.84 | 53 | 30.80 | DLPFC | Sudden medical condition |
| MD | M | NS | 7.20 | 2.98 | 6.40 | 56 | 21.50 | DLPFC | Sudden medical condition |
| MD | M | NS | 8.50 | 3.38 | 6.41 | 59 | 25.50 | DLPFC | Sudden medical condition |
| BP | F | Suicide | 7.9 | 3.11 | 7.13 | 34 | 25.50 | DLPFC | Suicide |
| MD | M | Suicide | 7.2 | 3.3 | 6.96 | 70 | 30.00 | DLPFC | Suicide |
| MD | M | Suicide | 7.7 | 2.62 | 6.76 | 46 | 26.50 | DLPFC | Suicide |
| MD | F | Suicide | 8 | 3.39 | 6.58 | 52 | 19.50 | DLPFC | Suicide |
| MD | M | Suicide | 7.8 | 3.64 | 6.69 | 36 | 13.00 | DLPFC | Suicide |
| MD | M | Suicide | 8.3 | 3.41 | 6.41 | 33 | 27.50 | DLPFC | Suicide |
| MD | F | Suicide | 8.4 | 3.02 | 7.39 | 46 | 28.00 | DLPFC | Suicide |
| MD | M | Suicide | 8.1 | 3.11 | 6.99 | 40 | 23.00 | DLPFC | Suicide |
| BP | M | Suicide | 7.9 | 3.11 | 6.84 | 50 | 25.50 | DLPFC | Suicide |
| BP | M | Suicide | 8.2 | 3.17 | 7.18 | 39 | 23.50 | DLPFC | Suicide |
| MD | M | Suicide | 7.2 | 5.15 | 6.4 | 77 | 24.00 | DLPFC | Suicide |
| MD | M | Suicide | 8.1 | 2.84 | 7.16 | 24 | 29.00 | DLPFC | Suicide |
| MD | M | Suicide | 7 | 3.32 | 6.51 | 34 | 25.30 | DLPFC | Suicide |
| MD | M | Suicide | 8.5 | 3.57 | 6.41 | 48 | 22.20 | DLPFC | Suicide |
| MD | M | Suicide | 7.8 | 3.28 | 7 | 31 | 27.20 | DLPFC | Suicide |
| MD | M | NS | 8.40 | 2.98 | 6.91 | 46 | 27.00 | Nacc | Sudden medical condition |
| BP | M | NS | 7.20 | 2.95 | 6.87 | 63 | 14.00 | Nacc | Accident |
| BP | M | NS | 8.40 | 3.05 | 6.99 | 59 | 15.50 | Nacc | Sudden medical condition |
| MD | M | NS | 7.60 | 3.16 | 7.05 | 66 | 32.00 | Nacc | Sudden medical condition |
| MD | F | NS | 7.50 | 3.18 | 7.06 | 44 | 25.00 | Nacc | Sudden medical condition |
| BP | F | NS | 8.20 | 2.68 | 6.55 | 59 | 27.00 | Nacc | Sudden medical condition |
| MD | M | NS | 8.10 | 2.94 | 6.84 | 50 | 30.80 | Nacc | Sudden medical condition |
| MD | M | NS | 8.20 | 2.53 | 6.41 | 48 | 25.50 | Nacc | Sudden medical condition |
| MD | M | Suicide | 7.90 | 2.99 | 6.93 | 58 | 24.00 | Nacc | Suicide |
| MD | M | Suicide | 8.40 | 2.92 | 6.79 | 39 | 27.50 | Nacc | Suicide |
| MD | M | Suicide | 7.60 | 3.47 | 7.25 | 47 | 29.00 | Nacc | Suicide |
| BP | F | Suicide | 7.90 | 3.12 | 7.00 | 36 | 25.50 | Nacc | Suicide |
| MD | F | Suicide | 8.00 | 3.22 | 7.16 | 53 | 19.50 | Nacc | Suicide |
| MD | M | Suicide | 7.80 | 2.95 | 6.96 | 56 | 13.00 | Nacc | Suicide |
| MD | F | Suicide | 8.40 | 2.88 | 6.76 | 46 | 28.00 | Nacc | Suicide |
| MD | M | Suicide | 8.10 | 2.68 | 6.58 | 52 | 23.00 | Nacc | Suicide |
| BP | M | Suicide | 7.90 | 2.85 | 6.69 | 36 | 25.50 | Nacc | Suicide |
| MD | M | Suicide | 8.10 | 3.29 | 7.18 | 39 | 29.00 | Nacc | Suicide |
| MD | M | Suicide | 8.50 | 2.59 | 6.51 | 34 | 22.20 | Nacc | Suicide |
| MD | M | Suicide | 9.20 | 2.24 | 6.33 | 29 | 19.00 | Nacc | Suicide |
| MD | M | Suicide | 7.80 | 3.09 | 7.00 | 31 | 27.20 | Nacc | Suicide |

ACC: anterior cingulate cortex; DLPFC: dorsolateral prefrontal cortex; NAcc: nucleus accumbens; RIN: Agilent 2100 RNA Integrity Number; MDD: major depressive disorder; BD: bipolar disorder; NS: non suicide; MOD: method of death.
