# Supplementary material for: Gene Expression Changes in the Prefrontal Cortex, Anterior Cingulate Cortex and Nucleus Accumbens of Mood Disorders Subjects That Committed Suicide
Source: PLoS One. 2012 Apr 30;7(4):e35367. doi: 10.1371/journal.pone.0035367 (PMC3340369; doi:10.1371/journal.pone.0035367)
Supplement: Table S2 — Complete demographic variables for the subjects included in the confirmation qPCR analysis of the DLPFC, ACC and Nacc. (MDD: major depressive disorder; BD: bipolar disorder; NS: non suicide; PMI: post-mortem interval; MOD: method of death). (DOC) [file pone.0035367.s002.doc]

**Supporting table 2.** Complete demographic variables for the subjects included in the confirmation qPCR analysis of the ACC, DLPFC and NAcc.

| Diagnosis | Group | Gender | Status | RIN | pH | Age | PMI | Region | MOD |
| --- | --- | --- | --- | --- | --- | --- | --- | --- | --- |
| Control | Control | M | NS | 7.9 | 7.0 | 18.0 | 22.0 | ACC | Accident |
| Control | Control | M | NS | 7.7 | 6.9 | 55.0 | 15.0 | ACC | Sudden medical condition |
| Control | Control | M | NS | 7.8 | 7.0 | 58.0 | 26.0 | ACC | Sudden medical condition |
| Control | Control | F | NS | 7.8 | 7.0 | 60.0 | 24.0 | ACC | Sudden medical condition |
| Control | Control | M | NS | 8.4 | 7.0 | 32.0 | 26.0 | ACC | Accident |
| Control | Control | M | NS | 7.8 | 6.9 | 44.0 | 23.0 | ACC | Sudden medical condition |
| Control | Control | M | NS | 8.8 | 6.8 | 39.0 | 18.2 | ACC | Sudden medical condition |
| Control | Control | M | NS | 7.2 | 7.0 | 39.0 | 30.0 | ACC | Accident |
| Control | Control | M | NS | 8.4 | 7.0 | 41.0 | 22.5 | ACC | Sudden medical condition |
| Control | Control | M | NS | 8.2 | 6.9 | 65.0 | 13.5 | ACC | Sudden medical condition |
| Control | Control | F | NS | 6.6 | 7.1 | 45.0 | 16.0 | ACC | Accident |
| Control | Control | M | NS | 7.0 | 7.3 | 63.0 | 22.7 | ACC | Sudden medical condition |
| Control | Control | M | NS | 7.0 | 7.1 | 56.0 | 20.3 | ACC | Accident |
| Control | Control | M | NS | 7.4 | 7.2 | 30.0 | 18.2 | ACC | Sudden medical condition |
| Control | Control | F | NS | 10.0 | 7.1 | 62.0 | 20.0 | ACC | Accident |
| Control | Control | M | NS | 8.4 | 6.5 | 52.0 | 18.8 | ACC | Sudden medical condition |
| Control | Control | M | NS | 8.4 | 6.5 | 63.0 | 11.0 | ACC | Sudden medical condition |
| Control | Control | M | NS | 9.2 | 7.1 | 64.0 | 10.5 | ACC | Sudden medical condition |
| MD | Mood-NS | M | NS | 7.9 | 6.8 | 52.0 | 16.0 | ACC | Sudden medical condition |
| MD | Mood-NS | M | NS | 8.0 | 6.9 | 46.0 | 27.0 | ACC | Sudden medical condition |
| MD | Mood-NS | M | NS | 8.5 | 7.2 | 63.0 | 28.5 | ACC | Sudden medical condition |
| MD | Mood-NS | M | NS | 7.5 | 7.1 | 66.0 | 32.0 | ACC | Sudden medical condition |
| MD | Mood-NS | F | NS | 7.5 | 7.1 | 44.0 | 25.0 | ACC | Sudden medical condition |
| BP | Mood-NS | F | NS | 8.8 | 6.6 | 59.0 | 27.0 | ACC | Sudden medical condition |
| MD | Mood-NS | M | NS | 9.9 | 6.8 | 50.0 | 30.8 | ACC | Sudden medical condition |
| MD | Mood-S | M | Suicide | 7.5 | 6.9 | 58.0 | 24.0 | ACC | Suicide |
| MD | Mood-S | M | Suicide | 7.1 | 7.2 | 49.0 | 27.0 | ACC | Suicide |
| MD | Mood-S | M | Suicide | 8.7 | 7.3 | 47.0 | 29.0 | ACC | Suicide |
| MD | Mood-S | M | Suicide | 7.0 | 7.1 | 28.0 | 26.5 | ACC | Suicide |
| MD | Mood-S | F | Suicide | 8.3 | 7.2 | 53.0 | 19.5 | ACC | Suicide |
| MD | Mood-S | M | Suicide | 7.9 | 7.0 | 56.0 | 13.0 | ACC | Suicide |
| MD | Mood-S | M | Suicide | 7.8 | 7.1 | 34.0 | 27.5 | ACC | Suicide |
| MD | Mood-S | M | Suicide | 10.0 | 7.0 | 40.0 | 24.0 | ACC | Suicide |
| MD | Mood-S | M | Suicide | 8.6 | 6.5 | 34.0 | 22.2 | ACC | Suicide |
| MD | Mood-S | M | Suicide | 8.8 | 7.0 | 31.0 | 27.2 | ACC | Suicide |
| Control | Control | M | NS | 6.5 | 7.0 | 18.0 | 22.0 | DLPFC | Accident |
| Control | Control | M | NS | 6.6 | 6.6 | 19.0 | 14.0 | DLPFC | Accident |
| Control | Control | M | NS | 7.4 | 6.9 | 55.0 | 15.0 | DLPFC | Sudden medical condition |
| Control | Control | M | NS | 7.6 | 7.0 | 58.0 | 26.0 | DLPFC | Sudden medical condition |
| Control | Control | F | NS | 7.0 | 7.0 | 60.0 | 24.0 | DLPFC | Sudden medical condition |
| Control | Control | M | NS | 7.9 | 7.0 | 32.0 | 26.0 | DLPFC | Accident |
| Control | Control | M | NS | 8.2 | 6.8 | 39.0 | 18.2 | DLPFC | Sudden medical condition |
| Control | Control | F | NS | 7.9 | 6.3 | 47.0 | 19.5 | DLPFC | Sudden medical condition |
| Control | Control | M | NS | 8.5 | 6.8 | 35.0 | 32.0 | DLPFC | Sudden medical condition |
| Control | Control | M | NS | 8.4 | 7.0 | 39.0 | 30.0 | DLPFC | Accident |
| Control | Control | M | NS | 8.3 | 7.0 | 41.0 | 22.5 | DLPFC | Sudden medical condition |
| Control | Control | M | NS | 8.1 | 6.9 | 65.0 | 13.5 | DLPFC | Sudden medical condition |
| Control | Control | M | NS | 7.0 | 7.1 | 40.0 | 36.5 | DLPFC | Sudden medical condition |
| Control | Control | M | NS | 8.2 | 6.9 | 66.0 | 18.0 | DLPFC | Sudden medical condition |
| Control | Control | M | NS | 7.8 | 7.2 | 55.0 | 31.5 | DLPFC | Sudden medical condition |
| Control | Control | M | NS | 6.8 | 7.1 | 69.0 | 25.8 | DLPFC | Sudden medical condition |
| Control | Control | M | NS | 7.3 | 7.3 | 63.0 | 22.7 | DLPFC | Sudden medical condition |
| Control | Control | M | NS | 7.5 | 7.1 | 56.0 | 20.3 | DLPFC | Accident |
| Control | Control | M | NS | 7.6 | 7.0 | 67.0 | 15.0 | DLPFC | Sudden medical condition |
| Control | Control | M | NS | 8.7 | 7.2 | 30.0 | 18.2 | DLPFC | Sudden medical condition |
| Control | Control | M | NS | 8.3 | 7.0 | 32.0 | 10.0 | DLPFC | Accident |
| Control | Control | F | NS | 7.9 | 7.1 | 62.0 | 20.0 | DLPFC | Accident |
| Control | Control | M | NS | 7.6 | 6.6 | 58.0 | 21.0 | DLPFC | Sudden medical condition |
| Control | Control | M | NS | 8.1 | 6.5 | 52.0 | 18.8 | DLPFC | Sudden medical condition |
| Control | Control | M | NS | 8.8 | 6.4 | 59.0 | 8.5 | DLPFC | Sudden medical condition |
| Control | Control | M | NS | 8.3 | 6.5 | 63.0 | 11.0 | DLPFC | Sudden medical condition |
| Control | Control | M | NS | 8.3 | 7.1 | 64.0 | 10.5 | DLPFC | Sudden medical condition |
| MD | Mood-NS | M | NS | 6.1 | 6.8 | 52.0 | 16.0 | DLPFC | Sudden medical condition |
| MD | Mood-NS | M | NS | 8.4 | 6.9 | 46.0 | 27.0 | DLPFC | Sudden medical condition |
| BP | Mood-NS | M | NS | 7.2 | 6.9 | 63.0 | 14.0 | DLPFC | Accident |
| MD | Mood-NS | M | NS | 7.5 | 7.0 | 35.0 | 24.8 | DLPFC | Accident |
| BP | Mood-NS | M | NS | 8.4 | 7.0 | 59.0 | 15.5 | DLPFC | Sudden medical condition |
| BP | Mood-NS | F | NS | 8.5 | 6.7 | 34.0 | 21.0 | DLPFC | Accident |
| MD | Mood-NS | M | NS | 8.1 | 7.2 | 63.0 | 28.5 | DLPFC | Sudden medical condition |
| MD | Mood-NS | M | NS | 7.6 | 7.1 | 66.0 | 32.0 | DLPFC | Sudden medical condition |
| BP | Mood-NS | F | NS | 8.2 | 6.6 | 59.0 | 27.0 | DLPFC | Sudden medical condition |
| MD | Mood-NS | M | NS | 8.1 | 6.8 | 50.0 | 30.8 | DLPFC | Sudden medical condition |
| MD | Mood-NS | M | NS | 8.2 | 6.4 | 48.0 | 25.5 | DLPFC | Sudden medical condition |
| MD | Mood-S | F | Suicide | 7.1 | 7.1 | 72.0 | 21.0 | DLPFC | Suicide |
| MD | Mood-S | M | Suicide | 7.9 | 6.9 | 58.0 | 24.0 | DLPFC | Suicide |
| BP | Mood-S | F | Suicide | 7.4 | 6.8 | 56.0 | 29.0 | DLPFC | Suicide |
| MD | Mood-S | F | Suicide | 7.8 | 7.0 | 48.0 | 37.0 | DLPFC | Suicide |
| MD | Mood-S | M | Suicide | 7.9 | 7.2 | 49.0 | 27.0 | DLPFC | Suicide |
| BP | Mood-S | M | Suicide | 7.9 | 7.1 | 52.0 | 28.0 | DLPFC | Suicide |
| BP | Mood-S | M | Suicide | 8.0 | 6.9 | 32.0 | 23.8 | DLPFC | Suicide |
| MD | Mood-S | M | Suicide | 7.6 | 7.3 | 47.0 | 29.0 | DLPFC | Suicide |
| BP | Mood-S | F | Suicide | 7.9 | 7.0 | 36.0 | 25.5 | DLPFC | Suicide |
| MD | Mood-S | M | Suicide | 7.7 | 7.1 | 28.0 | 26.5 | DLPFC | Suicide |
| MD | Mood-S | F | Suicide | 8.0 | 7.2 | 53.0 | 19.5 | DLPFC | Suicide |
| MD | Mood-S | M | Suicide | **8.3** | 7.1 | 34.0 | 27.5 | DLPFC | Suicide |
| MD | Mood-S | F | Suicide | 8.4 | 6.8 | 46.0 | 28.0 | DLPFC | Suicide |
| MD | Mood-S | M | Suicide | 8.1 | 6.6 | 52.0 | 23.0 | DLPFC | Suicide |
| BP | Mood-S | M | Suicide | 7.9 | 6.7 | 36.0 | 25.5 | DLPFC | Suicide |
| MD | Mood-S | M | Suicide | 7.2 | 7.0 | 40.0 | 24.0 | DLPFC | Suicide |
| MD | Mood-S | M | Suicide | 8.1 | 7.2 | 39.0 | 29.0 | DLPFC | Suicide |
| MD | Mood-S | M | Suicide | 8.5 | 6.5 | 34.0 | 22.2 | DLPFC | Suicide |
| MD | Mood-S | M | Suicide | 9.2 | 6.3 | 29.0 | 19.0 | DLPFC | Suicide |
| MD | Mood-S | M | Suicide | 7.8 | 7.0 | 31.0 | 27.2 | DLPFC | Suicide |
| Control | Control | M | NS |  | 7.0 | 32.0 | 26.0 | NACC | Accident |
| Control | Control | M | NS |  | 6.9 | 44.0 | 23.0 | NACC | Sudden medical condition |
| Control | Control | M | NS |  | 6.8 | 39.0 | 18.2 | NACC | Sudden medical condition |
| Control | Control | M | NS |  | 7.0 | 39.0 | 30.0 | NACC | Accident |
| Control | Control | M | NS |  | 7.0 | 41.0 | 22.5 | NACC | Sudden medical condition |
| Control | Control | F | NS |  | 7.1 | 45.0 | 16.0 | NACC | Accident |
| Control | Control | M | NS |  | 7.2 | 30.0 | 18.2 | NACC | Sudden medical condition |
| Control | Control | M | NS |  | 7.0 | 32.0 | 10.0 | NACC | Accident |
| Control | Control | M | NS |  | 6.6 | 58.0 | 21.0 | NACC | Sudden medical condition |
| Control | Control | M | NS |  | 6.5 | 52.0 | 18.8 | NACC | Sudden medical condition |
| MD | Mood-NS | M | NS |  | 6.8 | 52.0 | 16.0 | NACC | Sudden medical condition |
| MD | Mood-NS | M | NS |  | 6.9 | 46.0 | 27.0 | NACC | Sudden medical condition |
| MD | Mood-NS | M | NS |  | 7.0 | 35.0 | 24.8 | NACC | Accident |
| BP | Mood-NS | M | NS |  | 7.0 | 59.0 | 15.5 | NACC | Sudden medical condition |
| MD | Mood-NS | F | NS |  | 7.1 | 44.0 | 25.0 | NACC | Sudden medical condition |
| BP | Mood-NS | F | NS |  | 6.6 | 59.0 | 27.0 | NACC | Sudden medical condition |
| MD | Mood-NS | M | NS |  | 6.8 | 50.0 | 30.8 | NACC | Sudden medical condition |
| MD | Mood-S | M | Suicide |  | 6.9 | 58.0 | 24.0 | NACC | Suicide |
| BP | Mood-S | F | Suicide |  | 6.8 | 56.0 | 29.0 | NACC | Suicide |
| BP | Mood-S | M | Suicide |  | 7.1 | 52.0 | 28.0 | NACC | Suicide |
| BP | Mood-S | M | Suicide |  | 6.9 | 32.0 | 23.8 | NACC | Suicide |
| MD | Mood-S | M | Suicide |  | 6.8 | 39.0 | 27.5 | NACC | Suicide |
| MD | Mood-S | M | Suicide |  | 7.3 | 47.0 | 29.0 | NACC | Suicide |
| BP | Mood-S | M | Suicide |  | 6.9 | 49.0 | 29.5 | NACC | Suicide |
| BP | Mood-S | F | Suicide |  | 7.0 | 36.0 | 25.5 | NACC | Suicide |
| MD | Mood-S | F | Suicide |  | 7.2 | 53.0 | 19.5 | NACC | Suicide |
| MD | Mood-S | M | Suicide |  | 6.9 | 56.0 | 13.0 | NACC | Suicide |
| MD | Mood-S | M | Suicide |  | 7.1 | 34.0 | 27.5 | NACC | Suicide |
| MD | Mood-S | M | Suicide |  | 6.6 | 52.0 | 23.0 | NACC | Suicide |
| BP | Mood-S | M | Suicide |  | 6.7 | 36.0 | 25.5 | NACC | Suicide |

ACC: anterior cingulate cortex; DLPFC: dorsolateral prefrontal cortex; NAcc: nucleus accumbens; RIN: Agilent 2100 RNA Integrity Number; MDD: major depressive disorder; BD: bipolar disorder; NS: non suicide; MOD: method of death.
