# Supplementary material for: Gene Expression Changes in the Prefrontal Cortex, Anterior Cingulate Cortex and Nucleus Accumbens of Mood Disorders Subjects That Committed Suicide
Source: PLoS One. 2012 Apr 30;7(4):e35367. doi: 10.1371/journal.pone.0035367 (PMC3340369; doi:10.1371/journal.pone.0035367)
Supplement: Table S3 — Differentially expressed probe sets between suicide and non-suicide mood disorder subjects in the DLPFC. (DOC) [file pone.0035367.s003.doc]

**Supporting Table 3.** Differentially expressed probe sets between suicide and non suicide mood disorder subjects in the DLPFC.

| **Probeset** | **Gene Title** | **Symbol** | **Cytoband** | **p-value** | **NS** | **Suicide** | **FC** |
| --- | --- | --- | --- | --- | --- | --- | --- |
| 207135_at | 5-hydroxytryptamine (serotonin) receptor 2A | HTR2A | 13q14-q21 | 0.00022 | 10.06 | 9.70 | -1.28 |
| 223024_at | adaptor-related protein complex 1, mu 1 subunit | AP1M1 | 19p13.12 | 0.00774 | 9.68 | 9.32 | -1.28 |
| 235049_at | adenylate cyclase 1 (brain) | ADCY1 | 7p13-p12 | 0.00504 | 10.10 | 9.78 | -1.25 |
| 229704_at | Androgen-induced proliferation inhibitor | APRIN | 13q12.3 | 0.00690 | 8.49 | 9.83 | 2.53 |
| 218608_at | ATPase type 13A2 | ATP13A2 | 1p36 | 0.00168 | 9.22 | 8.79 | -1.35 |
| 206866_at | cadherin 4, type 1, R-cadherin (retinal) | CDH4 | 20q13.3 | 0.00329 | 8.07 | 7.74 | -1.26 |
| 211075_s_at | CD47 molecule | CD47 | 3q13.1-q13.2 | 0.00506 | 7.50 | 7.96 | 1.38 |
| 228748_at | CD59 molecule, complement regulatory protein | CD59 | 11p13 | 0.00601 | 6.17 | 5.80 | -1.30 |
| 239536_at | Chromosome 1 open reading frame 56 | C1orf56 | --- | 0.00773 | 5.43 | 5.79 | 1.28 |
| 228658_at | chromosome 22 open reading frame 35 | C22orf35 | 22q12.1 | 0.00183 | 10.21 | 9.53 | -1.61 |
| 227455_at | chromosome 6 open reading frame 136 | C6orf136 | 6p21.33 | 0.00800 | 8.89 | 8.56 | -1.26 |
| 203078_at | cullin 2 | CUL2 | 10p11.21 | 0.00395 | 5.70 | 5.27 | -1.35 |
| 206752_s_at | DNA fragmentation factor, 40kDa, beta polypeptide (caspase-activated DNase) | DFFB | 1p36.3 | 0.00228 | 6.08 | 5.67 | -1.33 |
| 208335_s_at | Duffy blood group, chemokine receptor | DARC | 1q21-q22 | 0.00742 | 7.24 | 6.85 | -1.31 |
| 226145_s_at | Fraser syndrome 1 | FRAS1 | 4q21.21 | 0.00528 | 7.10 | 6.36 | -1.68 |
| 220108_at | guanine nucleotide binding protein (G protein), alpha 14 | GNA14 | 9q21 | 0.00160 | 7.24 | 7.60 | 1.28 |
| 225245_x_at | H2A histone family, member J | H2AFJ | 12p12 | 0.00962 | 6.88 | 7.24 | 1.28 |
| 215071_s_at | histone cluster 1, H2ac | HIST1H2AC | 6p21.3 | 0.00502 | 6.18 | 7.31 | 2.20 |
| 227778_at | hypothetical protein KIAA1833 | KIAA1833 | 8q24.3 | 0.00928 | 8.19 | 7.86 | -1.26 |
| 213703_at | hypothetical protein LOC150759 | LOC150759 | 2q11.2 | 0.00803 | 7.08 | 6.75 | -1.26 |
| 227677_at | Janus kinase 3 (a protein tyrosine kinase, leukocyte) | JAK3 | 19p13.1 | 0.00069 | 7.64 | 7.30 | -1.26 |
| 224307_x_at | membrane associated guanylate kinase, WW and PDZ domain containing 3 | MAGI3 | 1p12-p11.2 | 0.00416 | 6.41 | 6.02 | -1.31 |
| 222447_at | methyltransferase like 9 | METTL9 | 16p13-p12 | 0.00335 | 9.62 | 9.22 | -1.31 |
| 213045_at | microtubule associated serine/threonine kinase 3 | MAST3 | 19p13.11 | 0.00433 | 10.19 | 9.80 | -1.31 |
| 235783_at | mRNA turnover 4 homolog (S. cerevisiae) | MRTO4 | 1p36.13 | 0.00078 | 7.23 | 6.90 | -1.25 |
| 204684_at | neuronal pentraxin I | NPTX1 | 17q25.1-q25.2 | 0.00139 | 10.30 | 9.94 | -1.28 |
| 219862_s_at | nuclear prelamin A recognition factor | NARF | 17q25.3 | 0.00400 | 8.67 | 8.32 | -1.27 |
| 210004_at | oxidized low density lipoprotein (lectin-like) receptor 1 | OLR1 | 12p13.2-p12.3 | 0.00673 | 5.92 | 5.49 | -1.35 |
| 202861_at | period homolog 1 (Drosophila) | PER1 | 17p13.1-p12 | 0.00562 | 8.77 | 8.45 | -1.25 |
| 226823_at | phosphatase and actin regulator 4 | PHACTR4 | 1p35.3 | 0.00546 | 7.76 | 8.19 | 1.34 |
| 210910_s_at | POM (POM121 homolog, rat) and ZP3 fusion | POMZP3 | 7q11.23 | 0.00231 | 7.24 | 6.32 | -1.88 |
| 227192_at | proline-rich transmembrane protein 2 | PRRT2 | 16p11.2 | 0.00596 | 9.13 | 8.75 | -1.30 |
| 206691_s_at | protein disulfide isomerase family A, member 2 | PDIA2 | 16p13.3 | 0.00704 | 7.59 | 7.22 | -1.29 |
| 219142_at | RAS-like, family 11, member B | RASL11B | 4q12 | 0.00666 | 8.75 | 8.22 | -1.45 |
| 1557688_at | Rho GTPase activating protein 26 | ARHGAP26 | --- | 0.00477 | 7.76 | 7.42 | -1.26 |
| 223044_at | solute carrier family 40 (iron-regulated transporter), member 1 | SLC40A1 | 2q32 | 0.00297 | 5.70 | 6.22 | 1.44 |
| 209437_s_at | spondin 1, extracellular matrix protein | SPON1 | 11p15.2 | 0.00905 | 6.92 | 7.38 | 1.37 |
| 206020_at | suppressor of cytokine signaling 6 | SOCS6 | 18q22.2 | 0.00246 | 4.81 | 5.15 | 1.26 |
| 200629_at | tryptophanyl-tRNA synthetase | WARS | 14q32.31 | 0.00290 | 9.24 | 8.84 | -1.31 |
| 1560648_s_at | TSPY-like 1 | TSPYL1 | 6q22-q23 | 0.00400 | 8.64 | 8.19 | -1.36 |
| 204148_s_at | zona pellucida glycoprotein 3 (sperm receptor) | ZP3 | 7q11.23 | 0.00242 | 7.30 | 6.34 | -1.94 |
