# Supplementary material for: Gene Expression Changes in the Prefrontal Cortex, Anterior Cingulate Cortex and Nucleus Accumbens of Mood Disorders Subjects That Committed Suicide
Source: PLoS One. 2012 Apr 30;7(4):e35367. doi: 10.1371/journal.pone.0035367 (PMC3340369; doi:10.1371/journal.pone.0035367)
Supplement: Table S4 — Differentially expressed probe sets between suicide and non-suicide mood disorder subjects in the ACC (139). (DOC) [file pone.0035367.s004.doc]

**Supporting Table 4.** Differentially expressed probe sets between suicide and non suicide mood disorder subjects in the ACC (139).

| Probeset | Gene Title | Gene Symbol | Cytoband | p-value | NS | Suicide | FC |
| --- | --- | --- | --- | --- | --- | --- | --- |
| 217520_x_at | Similar to programmed cell death 6 interacting protein | LOC731884 | --- | 0.00608 | 8.60 | 9.09 | 1.40 |
| 229901_at | zinc finger protein 488 | ZNF488 | 10q11.22 | 0.00548 | 8.51 | 8.86 | 1.27 |
| 212500_at | 2-aminoethanethiol (cysteamine) dioxygenase | ADO | 10q21.2 | 0.00890 | 9.73 | 10.19 | 1.38 |
| 228839_s_at | hypothetical gene supported by AF064843; AK025716 | LOC439994 | 10q22.3 | 0.00032 | 8.27 | 8.68 | 1.32 |
| 203571_s_at | chromosome 10 open reading frame 116 | C10orf116 | 10q23.2 | 0.00116 | 9.26 | 8.40 | -1.80 |
| 218322_s_at | acyl-CoA synthetase long-chain family member 5 | ACSL5 | 10q25.1-q25.2 | 0.00834 | 7.86 | 7.26 | -1.52 |
| 221840_at | protein tyrosine phosphatase, receptor type, E | PTPRE | 10q26 | 0.00094 | 8.98 | 9.32 | 1.27 |
| 211617_at | aldolase A, fructose-bisphosphate pseudogene 2 | ALDOAP2 | 10q26.13 | 0.00188 | 6.95 | 6.52 | -1.35 |
| 1554397_s_at | UEV and lactate/malate dehyrogenase domains | UEVLD | 11p15.1 | 0.00537 | 6.71 | 7.18 | 1.38 |
| 212590_at | related RAS viral (r-ras) oncogene homolog 2 | RRAS2 | 11p15.2 | 0.00193 | 7.19 | 6.84 | -1.28 |
| 1558014_s_at | fatty acyl CoA reductase 1 | FAR1 | 11p15.2 | 0.00760 | 6.50 | 7.39 | 1.85 |
| 214133_at | mucin 6, oligomeric mucus/gel | MUC6 | 11p15.5-p15.4 | 0.00054 | 6.94 | 6.55 | -1.30 |
| 219025_at | CD248 molecule, endosialin | CD248 | 11q13 | 0.00711 | 8.20 | 7.88 | -1.25 |
| 219587_at | tetratricopeptide repeat domain 12 | TTC12 | 11q23.1 | 0.00429 | 6.01 | 6.36 | 1.28 |
| 236359_at | sodium channel, voltage-gated, type IV, beta | SCN4B | 11q23.3 | 0.00918 | 9.70 | 10.49 | 1.74 |
| 230320_at | transforming growth factor beta regulator 1 | TBRG1 | 11q24.2 | 0.00063 | 8.60 | 8.96 | 1.28 |
| 225819_at | transforming growth factor beta regulator 1 | TBRG1 | 11q24.2 | 0.00079 | 7.85 | 8.22 | 1.29 |
| 226318_at | transforming growth factor beta regulator 1 | TBRG1 | 11q24.2 | 0.00205 | 8.34 | 8.67 | 1.26 |
| 206989_s_at | splicing factor, arginine/serine-rich 2, interacting protein | SFRS2IP | 12q12-q13.11 | 0.00276 | 7.64 | 8.23 | 1.50 |
| 237701_at | chromosome 12 open reading frame 54 | C12orf54 | 12q13.11 | 0.00367 | 7.44 | 6.98 | -1.38 |
| 234133_s_at | hypothetical protein LOC728543 | LOC728543 | 12q24.13 | 0.00062 | 7.17 | 6.85 | -1.25 |
| 219372_at | intraflagellar transport 81 homolog (Chlamydomonas) | IFT81 | 12q24.13 | 0.00236 | 7.03 | 7.45 | 1.34 |
| 205461_at | RAB35, member RAS oncogene family | RAB35 | 12q24.31 | 0.00054 | 5.84 | 6.36 | 1.43 |
| 243429_at | hypothetical protein LOC100130979 | LOC100130979 | 13q12.11 | 0.00172 | 8.19 | 7.83 | -1.28 |
| 204085_s_at | ceroid-lipofuscinosis, neuronal 5 | CLN5 | 13q21.1-q32 | 0.00371 | 5.46 | 6.01 | 1.47 |
| 214276_at | Kruppel-like factor 12 | KLF12 | 13q22 | 0.00551 | 6.41 | 6.07 | -1.27 |
| 223254_s_at | KIAA1333 | KIAA1333 | 14q12 | 0.00065 | 7.55 | 8.16 | 1.53 |
| 229761_at | LOC440173 | LOC440173 | 14q12 | 0.00184 | 6.84 | 6.47 | -1.29 |
| 215495_s_at | sterile alpha motif domain containing 4A | SAMD4A | 14q22.2 | 0.00802 | 5.52 | 5.14 | -1.30 |
| 219757_s_at | chromosome 14 open reading frame 101 | C14orf101 | 14q23.1 | 0.00675 | 7.83 | 8.26 | 1.35 |
| 233208_x_at | cleavage and polyadenylation specific factor 2, 100kDa | CPSF2 | 14q31.1 | 0.00041 | 5.78 | 6.19 | 1.33 |
| 204797_s_at | echinoderm microtubule associated protein like 1 | EML1 | 14q32 | 0.00055 | 7.44 | 8.42 | 1.97 |
| 204796_at | echinoderm microtubule associated protein like 1 | EML1 | 14q32 | 0.00322 | 6.70 | 7.34 | 1.55 |
| 219840_s_at | T-cell leukemia/lymphoma 6 | TCL6 | 14q32.1 | 0.00912 | 7.24 | 6.91 | -1.26 |
| 217039_x_at | Immunoglobulin heavy constant mu | IGHG1 | 14q32.33 | 0.00820 | 8.72 | 8.24 | -1.39 |
| 214980_at | Ubiquitin protein ligase E3A | UBE3A | 15q11-q13 | 0.00695 | 7.78 | 6.88 | -1.87 |
| 202766_s_at | fibrillin 1 | FBN1 | 15q21.1 | 0.00186 | 7.04 | 7.43 | 1.30 |
| 244746_at | Sema domain, transmembrane domain (TM), and cytoplasmic domain, (semaphorin) 6D | SEMA6D | 15q21.1 | 0.00801 | 6.71 | 6.09 | -1.53 |
| 225327_at | KIAA1370 | KIAA1370 | 15q21.2 | 0.00052 | 7.90 | 8.28 | 1.30 |
| 227870_at | neighbor of Punc E11 | NOPE | 15q22.31 | 0.00879 | 7.12 | 6.32 | -1.75 |
| 206533_at | cholinergic receptor, nicotinic, alpha 5 | CHRNA5 | 15q24 | 0.00148 | 6.34 | 5.99 | -1.28 |
| 234725_s_at | sema domain, immunoglobulin domain (Ig), transmembrane domain (TM) and short cyt | SEMA4B | 15q25 | 0.00391 | 8.87 | 8.50 | -1.29 |
| 218033_s_at | stannin | SNN | 16p13 | 0.00962 | 8.44 | 8.97 | 1.44 |
| 206527_at | 4-aminobutyrate aminotransferase | ABAT | 16p13.2 | 0.00048 | 7.91 | 8.30 | 1.31 |
| 206461_x_at | metallothionein 1H | MT1H | 16q13 | 0.00052 | 11.57 | 10.85 | -1.65 |
| 204745_x_at | metallothionein 1G | MT1G | 16q13 | 0.00096 | 11.03 | 10.22 | -1.75 |
| 212859_x_at | metallothionein 1E | MT1E | 16q13 | 0.00117 | 11.55 | 10.85 | -1.62 |
| 217165_x_at | metallothionein 1F | MT1F | 16q13 | 0.00217 | 10.98 | 10.38 | -1.51 |
| 212185_x_at | metallothionein 2A | MT2A | 16q13 | 0.00255 | 12.27 | 11.52 | -1.68 |
| 213629_x_at | metallothionein 1F | MT1F | 16q13 | 0.00266 | 11.09 | 10.40 | -1.61 |
| 208581_x_at | metallothionein 1X | MT1X | 16q13 | 0.00311 | 11.27 | 10.39 | -1.83 |
| 202188_at | nucleoporin 93kDa | NUP93 | 16q13 | 0.00982 | 7.84 | 8.20 | 1.28 |
| 216336_x_at | metallothionein 1E /// metallothionein 1H /// metallothionein 1M /// metallothio | MT1E /// MT1H /// MT1M /// MT1P2 | 16q13 /// 1q43 | 0.00484 | 10.97 | 10.37 | -1.52 |
| 228784_at | ST3 beta-galactoside alpha-2,3-sialyltransferase 2 | ST3GAL2 | 16q22.1 | 0.00515 | 6.81 | 6.36 | -1.36 |
| 205045_at | A kinase (PRKA) anchor protein 10 | AKAP10 | 17p11.1 | 0.00165 | 6.09 | 6.44 | 1.27 |
| 239499_at | dynein, axonemal, heavy chain 2 | DNAH2 | 17p13.1 | 0.00295 | 6.19 | 6.51 | 1.25 |
| 227645_at | phosphoinositide-3-kinase, regulatory subunit 5 | PIK3R5 | 17p13.1 | 0.00346 | 6.98 | 6.62 | -1.29 |
| 227884_at | TAF15 RNA polymerase II, TATA box binding protein (TBP)-associated factor, 68kDa | TAF15 | 17q11.1-q11.2 | 0.00019 | 7.31 | 8.00 | 1.61 |
| 221249_s_at | family with sequence similarity 117, member A | FAM117A | 17q21.33 | 0.00738 | 7.96 | 7.54 | -1.34 |
| 207996_s_at | chromosome 18 open reading frame 1 | C18orf1 | 18p11.2 | 0.00725 | 9.38 | 9.79 | 1.33 |
| 1564190_x_at | zinc finger protein 519 | ZNF519 | 18p11.21 | 0.00403 | 7.82 | 8.33 | 1.42 |
| 216257_at | serpin peptidase inhibitor, clade B (ovalbumin), member 13 | SERPINB13 | 18q21.3-q22 | 0.00651 | 6.64 | 6.16 | -1.40 |
| 212574_x_at | chromosome 19 open reading frame 6 | C19orf6 | 19p13.3 | 0.00172 | 7.20 | 7.65 | 1.37 |
| 212575_at | chromosome 19 open reading frame 6 | C19orf6 | 19p13.3 | 0.00788 | 6.08 | 6.61 | 1.45 |
| 244176_at | hypothetical LOC401913 | LOC401913 | 19q13.11 | 0.00131 | 7.47 | 7.07 | -1.32 |
| 228305_at | zinc finger protein 565 | ZNF565 | 19q13.12 | 0.00004 | 7.51 | 8.25 | 1.67 |
| 204952_at | LY6/PLAUR domain containing 3 | LYPD3 | 19q13.31 | 0.00750 | 8.32 | 7.95 | -1.29 |
| 239381_at | kallikrein-related peptidase 7 | KLK7 | 19q13.33 | 0.00961 | 9.43 | 8.95 | -1.39 |
| 237002_at | Neurochondrin | NCDN | 1p34.3 | 0.00609 | 7.78 | 8.21 | 1.35 |
| 236498_s_at | chromosome 1 open reading frame 86 | C1orf86 | 1p36.33 | 0.00652 | 6.97 | 6.50 | -1.39 |
| 57082_at | low density lipoprotein receptor adaptor protein 1 | LDLRAP1 | 1p36-p35 | 0.00754 | 6.33 | 6.70 | 1.29 |
| 202251_at | PRP3 pre-mRNA processing factor 3 homolog (S. cerevisiae) | PRPF3 | 1q21.1 | 0.00768 | 6.72 | 7.20 | 1.40 |
| 207629_s_at | rho/rac guanine nucleotide exchange factor (GEF) 2 | ARHGEF2 | 1q21-q22 | 0.00192 | 8.38 | 8.71 | 1.26 |
| 1554783_s_at | rho/rac guanine nucleotide exchange factor (GEF) 2 | ARHGEF2 | 1q21-q22 | 0.00360 | 5.31 | 5.65 | 1.27 |
| 202220_at | KIAA0907 | KIAA0907 | 1q22 | 0.00643 | 9.21 | 9.60 | 1.32 |
| 226880_at | nuclear casein kinase and cyclin-dependent kinase substrate 1 | NUCKS1 | 1q32.1 | 0.00055 | 10.37 | 11.31 | 1.92 |
| 223884_at | opticin | OPTC | 1q32.1 | 0.00870 | 7.35 | 6.95 | -1.32 |
| 200843_s_at | glutamyl-prolyl-tRNA synthetase | EPRS | 1q41-q42 | 0.00671 | 9.75 | 10.09 | 1.26 |
| 211456_x_at | metallothionein 1 pseudogene 2 | MT1P2 | 1q43 | 0.00400 | 11.72 | 11.17 | -1.46 |
| 233396_s_at | CSRP2 binding protein | CSRP2BP | 20p11.23 | 0.00601 | 7.06 | 6.72 | -1.27 |
| 205289_at | bone morphogenetic protein 2 | BMP2 | 20p12 | 0.00151 | 5.18 | 5.52 | 1.26 |
| 218081_at | chromosome 20 open reading frame 27 | C20orf27 | 20p13 | 0.00821 | 8.10 | 8.58 | 1.39 |
| 225145_at | nuclear receptor coactivator 5 | NCOA5 | 20q12-q13.12 | 0.00554 | 7.40 | 7.79 | 1.31 |
| 209049_s_at | zinc finger, MYND-type containing 8 | ZMYND8 | 20q13.12 | 0.00243 | 8.83 | 9.37 | 1.46 |
| 232820_s_at | gametocyte specific factor 1-like | GTSF1L | 20q13.12 | 0.00513 | 7.87 | 7.55 | -1.25 |
| 218515_at | chromosome 21 open reading frame 66 | C21orf66 | 21q21.3 | 0.00596 | 8.35 | 8.82 | 1.39 |
| 205330_at | meningioma (disrupted in balanced translocation) 1 | MN1 | 22q11|22q12.1 | 0.00067 | 7.24 | 8.04 | 1.74 |
| 55081_at | MICAL-like 1 | MICALL1 | 22q13.1-q13.2 | 0.00465 | 5.68 | 6.12 | 1.35 |
| 212220_at | proteasome (prosome, macropain) activator subunit 4 | PSME4 | 2p16.2 | 0.00990 | 6.51 | 7.35 | 1.79 |
| 207478_at | similar to PRO2958 | LOC100128329 | 2p24.3 | 0.00501 | 5.53 | 5.20 | -1.25 |
| 219043_s_at | phosducin-like 3 | PDCL3 | 2q11.2 /// 3q12.3 | 0.00364 | 8.32 | 8.78 | 1.37 |
| 233079_at | c-mer proto-oncogene tyrosine kinase | MERTK | 2q14.1 | 0.00924 | 7.77 | 7.40 | -1.29 |
| 203664_s_at | polymerase (RNA) II (DNA directed) polypeptide D | POLR2D | 2q21 | 0.00402 | 6.45 | 6.96 | 1.42 |
| 226486_at | MTERF domain containing 2 | MTERFD2 | 2q37.3 | 0.00090 | 8.15 | 8.65 | 1.41 |
| 205855_at | zinc finger protein 197 | ZNF197 | 3p21 | 0.00101 | 7.42 | 7.09 | -1.26 |
| 1554411_at | catenin (cadherin-associated protein), beta 1, 88kDa | CTNNB1 | 3p21 | 0.00632 | 6.28 | 6.94 | 1.57 |
| 226074_at | protein phosphatase 1M (PP2C domain containing) | PPM1M | 3p21.1 | 0.00206 | 8.55 | 8.01 | -1.45 |
| 215203_at | golgi autoantigen, golgin subfamily a, 4 | GOLGA4 | 3p22-p21.3 | 0.00759 | 7.86 | 8.23 | 1.30 |
| 223211_at | 2-hydroxyacyl-CoA lyase 1 | HACL1 | 3p24.3 | 0.00832 | 7.69 | 8.03 | 1.27 |
| 220035_at | nucleoporin 210kDa | NUP210 | 3p25.1 | 0.00093 | 7.37 | 7.74 | 1.29 |
| 223444_at | SUMO1/sentrin specific peptidase 7 | SENP7 | 3q12 | 0.00212 | 7.31 | 8.04 | 1.66 |
| 235918_x_at | centrosomal protein 97kDa | CEP97 | 3q12.3 | 0.00965 | 8.56 | 9.15 | 1.51 |
| 229527_s_at | Oxysterol binding protein-like 11 | OSBPL11 | 3q21 | 0.00277 | 6.59 | 6.20 | -1.30 |
| 212058_at | U2-associated SR140 protein | SR140 | 3q23 | 0.00332 | 8.50 | 8.97 | 1.38 |
| 219871_at | hypothetical FLJ13197 | FLJ13197 | 4p14 | 0.00486 | 7.19 | 7.91 | 1.65 |
| 243668_at | Mediator complex subunit 28 | MED28 | 4p16 | 0.00496 | 8.59 | 8.20 | -1.31 |
| 227299_at | Cyclin I | CCNI | 4q21.1 | 0.00117 | 6.99 | 7.43 | 1.37 |
| 232119_at | synaptopodin 2 | SYNPO2 | 4q26 | 0.00211 | 4.96 | 5.28 | 1.25 |
| 213859_x_at | SWI/SNF related, matrix associated, actin dependent regulator of chromatin, subf | SMARCA5 | 4q31.1-q31.2 | 0.00470 | 5.90 | 6.25 | 1.27 |
| 204333_s_at | aspartylglucosaminidase | AGA | 4q32-q33 | 0.00650 | 6.20 | 7.01 | 1.75 |
| 1555495_a_at | serologically defined colon cancer antigen 10 | SDCCAG10 | 5q12.3 | 0.00995 | 9.14 | 9.56 | 1.33 |
| 235115_at | phosphodiesterase 8B | PDE8B | 5q14.1 | 0.00840 | 5.91 | 6.40 | 1.41 |
| 228686_at | hypothetical LOC644873 | FLJ33630 | 5q23.3 | 0.00400 | 6.33 | 7.11 | 1.72 |
| 206197_at | non-metastatic cells 5, protein expressed in (nucleoside-diphosphate kinase) | NME5 | 5q31 | 0.00534 | 8.67 | 9.24 | 1.49 |
| 202162_s_at | CCR4-NOT transcription complex, subunit 8 | CNOT8 | 5q31-q33 | 0.00781 | 8.30 | 8.79 | 1.40 |
| 219227_at | cyclin J-like | CCNJL | 5q33.3 | 0.00095 | 6.53 | 6.12 | -1.33 |
| 211223_at | PROP paired-like homeobox 1 | PROP1 | 5q35.3 | 0.00080 | 7.59 | 7.27 | -1.25 |
| 221457_s_at | butyrophilin-like 2 (MHC class II associated) | BTNL2 | 6p21.3 | 0.00848 | 7.58 | 7.04 | -1.45 |
| 211096_at | pre-B-cell leukemia homeobox 2 | PBX2 | 6p21.3 | 0.00946 | 7.77 | 7.44 | -1.26 |
| 203476_at | trophoblast glycoprotein | TPBG | 6q14-q15 | 0.00979 | 8.47 | 9.56 | 2.14 |
| 208653_s_at | CD164 molecule, sialomucin | CD164 | 6q21 | 0.00548 | 6.21 | 7.00 | 1.72 |
| 219974_x_at | enoyl Coenzyme A hydratase domain containing 1 | ECHDC1 | 6q22.33 | 0.00829 | 7.64 | 8.17 | 1.45 |
| 230109_at | phosphodiesterase 7B | PDE7B | 6q23-q24 | 0.00225 | 8.45 | 8.96 | 1.42 |
| 1554250_s_at | tripartite motif-containing 73 | TRIM73 | 7q11.23 | 0.00167 | 6.55 | 6.89 | 1.27 |
| 227265_at | fibrinogen-like 2 | FGL2 | 7q11.23 | 0.00755 | 5.98 | 6.68 | 1.62 |
| 202627_s_at | serpin peptidase inhibitor, clade E (nexin, plasminogen activator inhibitor type | SERPINE1 | 7q21.3-q22 | 0.00293 | 7.17 | 6.85 | -1.25 |
| 203065_s_at | caveolin 1, caveolae protein, 22kDa | CAV1 | 7q31.1 | 0.00689 | 7.55 | 8.07 | 1.43 |
| 231880_at | family with sequence similarity 40, member B | FAM40B | 7q32.1 | 0.00040 | 7.80 | 8.94 | 2.22 |
| 209341_s_at | inhibitor of kappa light polypeptide gene enhancer in B-cells, kinase beta | IKBKB | 8p11.2 | 0.00390 | 7.48 | 7.89 | 1.32 |
| 242041_at | centrosome and spindle pole associated protein 1 | CSPP1 | 8q13.2 | 0.00301 | 7.12 | 6.73 | -1.31 |
| 238122_at | RNA binding motif protein 12B | RBM12B | 8q22.1 | 0.00358 | 5.81 | 6.27 | 1.37 |
| 226942_at | PHD finger protein 20-like 1 | PHF20L1 | 8q24.22 | 0.00348 | 8.05 | 8.41 | 1.29 |
| 205407_at | reversion-inducing-cysteine-rich protein with kazal motifs | RECK | 9p13.3 | 0.00523 | 6.71 | 7.07 | 1.29 |
| 229568_at | MOB1, Mps One Binder kinase activator-like 2B (yeast) | MOBKL2B | 9p21.2 | 0.00855 | 7.17 | 8.34 | 2.25 |
| 209822_s_at | very low density lipoprotein receptor | VLDLR | 9p24 | 0.00590 | 7.29 | 7.71 | 1.34 |
| 230636_s_at | Kruppel-like factor 9 | KLF9 | 9q13 | 0.00064 | 5.57 | 6.00 | 1.35 |
| 204479_at | osteoclast stimulating factor 1 | OSTF1 | 9q13-q21.2 | 0.00962 | 7.52 | 8.01 | 1.40 |
| 210257_x_at | cullin 4B | CUL4B | Xq23 | 0.00013 | 6.31 | 6.74 | 1.35 |
| 225310_at | RNA binding motif protein, X-linked | RBMX | Xq26.3 | 0.00686 | 7.77 | 8.35 | 1.49 |
