# Supplementary material for: Gene Expression Changes in the Prefrontal Cortex, Anterior Cingulate Cortex and Nucleus Accumbens of Mood Disorders Subjects That Committed Suicide
Source: PLoS One. 2012 Apr 30;7(4):e35367. doi: 10.1371/journal.pone.0035367 (PMC3340369; doi:10.1371/journal.pone.0035367)
Supplement: Table S5 — Differentially expressed probe sets between suicide and non-suicide mood disorder subjects in the NAcc (146). (DOC) [file pone.0035367.s005.doc]

**Supporting Table 5.** Differentially expressed probe sets between suicide and non suicide mood disorder subjects in the NAcc (146).

| Probeset | Gene | Symbol | Cytoband | p-value | NS | Suicide | FC |
| --- | --- | --- | --- | --- | --- | --- | --- |
| 204395_s_at | G protein-coupled receptor kinase 5 | GRK5 | 10q24-qter | 0.0001 | 7.37 | 7.04 | -1.26 |
| 206461_x_at | metallothionein 1H | MT1H | 16q13 | 0.0001 | 10.28 | 9.83 | -1.36 |
| 1552326_a_at | coiled-coil domain containing 11 | CCDC11 | 18q21.1 | 0.0001 | 6.44 | 5.84 | -1.51 |
| 227274_at | synaptojanin 2 binding protein | SYNJ2BP | 14q24.2 | 0.0002 | 8.07 | 8.40 | 1.26 |
| 229993_at | Zinc finger, CCHC domain containing 3 | ZCCHC3 | 20p13-p12.2 | 0.0004 | 4.61 | 4.95 | 1.27 |
| 203465_at | mitochondrial ribosomal protein L19 | MRPL19 | 2q11.1-q11.2 | 0.0004 | 7.39 | 6.99 | -1.32 |
| 224777_s_at | platelet-activating factor acetylhydrolase, isoform Ib, beta subunit 30kDa | PAFAH1B2 | 11q23 | 0.0004 | 8.52 | 8.90 | 1.30 |
| 214085_x_at | GLI pathogenesis-related 1 | GLIPR1 | 12q21.2 | 0.0004 | 8.08 | 6.61 | -2.78 |
| 202269_x_at | guanylate binding protein 1, interferon-inducible, 67kDa | GBP1 | 1p22.2 | 0.0005 | 5.44 | 4.85 | -1.50 |
| 210032_s_at | sperm associated antigen 6 | SPAG6 | 10p12.2 | 0.0008 | 5.47 | 5.07 | -1.31 |
| 230040_at | ADAM metallopeptidase with thrombospondin type 1 motif, 18 | ADAMTS18 | 16q23 | 0.0008 | 5.07 | 5.76 | 1.62 |
| 219685_at | transmembrane protein 35 | TMEM35 | Xq22.1 | 0.0009 | 9.14 | 8.54 | -1.51 |
| 203185_at | Ras association (RalGDS/AF-6) domain family member 2 | RASSF2 | 20pter-p12.1 | 0.0009 | 8.04 | 8.78 | 1.67 |
| 219310_at | chromosome 20 open reading frame 39 | C20orf39 | 20p11.21 | 0.0010 | 6.69 | 7.09 | 1.32 |
| 221448_s_at | testis expressed 15 | TEX15 | 8p12 | 0.0010 | 5.22 | 4.82 | -1.31 |
| 1556599_s_at | cyclic AMP-regulated phosphoprotein, 21 kD | ARPP-21 /// LOC100130503 | 3p22.3 | 0.0011 | 4.38 | 4.82 | 1.36 |
| 213887_s_at | polymerase (RNA) II (DNA directed) polypeptide E, 25kDa | POLR2E | 19p13.3 | 0.0011 | 7.70 | 8.03 | 1.26 |
| 203934_at | kinase insert domain receptor (a type III receptor tyrosine kinase) | KDR | 4q11-q12 | 0.0011 | 4.79 | 5.17 | 1.30 |
| 215672_s_at | S-adenosylhomocysteine hydrolase-like 2 | AHCYL2 | 7q32.1 | 0.0011 | 5.66 | 5.99 | 1.26 |
| 205103_at | chromosome 1 open reading frame 61 | C1orf61 | 1q22 | 0.0012 | 11.62 | 11.94 | 1.25 |
| 234820_at | MAS1 oncogene-like | MAS1L | 6p21 | 0.0013 | 5.63 | 5.29 | -1.26 |
| 208641_s_at | ras-related C3 botulinum toxin substrate 1 (rho family, small GTP binding protei | RAC1 | 7p22 | 0.0014 | 8.64 | 9.15 | 1.42 |
| 204745_x_at | metallothionein 1G | MT1G | 16q13 | 0.0015 | 9.60 | 9.06 | -1.45 |
| 230807_at | coiled-coil domain containing 151 | CCDC151 | 19p13.2 | 0.0015 | 6.48 | 6.11 | -1.29 |
| 224751_at | PL-5283 protein | PL-5283 | 7q33 | 0.0015 | 6.13 | 6.53 | 1.32 |
| 202018_s_at | lactotransferrin | LTF | 3p21.31 | 0.0015 | 5.69 | 5.19 | -1.42 |
| 236085_at | calcyphosine-like | CAPSL | 5p13.2 | 0.0016 | 7.16 | 6.54 | -1.54 |
| 235252_at | Kinase suppressor of ras 1 | KSR1 | 17q11.1 | 0.0017 | 6.56 | 7.03 | 1.39 |
| 227605_at | small inducible cytokine subfamily E, member 1 (endothelial monocyte-activating) | SCYE1 | 4q24 | 0.0018 | 6.29 | 6.63 | 1.26 |
| 226424_at | calcyphosine | CAPS | 19p13.3 | 0.0019 | 6.72 | 6.22 | -1.42 |
| 231068_at | solute carrier family 47, member 2 | SLC47A2 | 17p11.2 | 0.0020 | 7.02 | 6.32 | -1.63 |
| 222068_s_at | leucine rich repeat containing 50 | LRRC50 | 16q24.1 | 0.0020 | 7.99 | 7.61 | -1.30 |
| 212423_at | zinc finger, CCHC domain containing 24 | ZCCHC24 | 10q22.3 | 0.0020 | 8.93 | 9.38 | 1.37 |
| 209163_at | cytochrome b-561 | CYB561 | 17q11-qter | 0.0021 | 7.86 | 7.53 | -1.26 |
| 204724_s_at | collagen, type IX, alpha 3 | COL9A3 | 20q13.3 | 0.0021 | 6.85 | 7.45 | 1.51 |
| 226753_at | family with sequence similarity 76, member B | FAM76B | 11q21 | 0.0021 | 5.09 | 5.52 | 1.35 |
| 231729_s_at | calcyphosine | CAPS | 19p13.3 | 0.0021 | 5.60 | 4.83 | -1.71 |
| 222116_s_at | TBC1 domain family, member 16 | TBC1D16 | 17q25.3 | 0.0022 | 7.07 | 7.58 | 1.42 |
| 214485_at | outer dense fiber of sperm tails 1 | ODF1 | 8q22.3 | 0.0022 | 6.99 | 6.67 | -1.25 |
| 226342_at | spectrin, beta, non-erythrocytic 1 | SPTBN1 | 2p21 | 0.0022 | 8.07 | 8.65 | 1.49 |
| 221944_at | Hypothetical LOC645644 | FLJ42627 | 16p13.3 | 0.0023 | 6.16 | 6.61 | 1.36 |
| 201280_s_at | disabled homolog 2, mitogen-responsive phosphoprotein (Drosophila) | DAB2 | 5p13 | 0.0023 | 5.38 | 5.75 | 1.30 |
| 228170_at | oligodendrocyte transcription factor 1 | OLIG1 | 21q22.11 | 0.0024 | 9.39 | 10.18 | 1.72 |
| 227046_at | solute carrier family 39 (metal ion transporter), member 11 | SLC39A11 | 17q24.3-q25.1 | 0.0024 | 6.27 | 6.63 | 1.29 |
| 1564064_a_at | ATPase, class VI, type 11B | ATP11B | 3q27 | 0.0026 | 5.58 | 5.21 | -1.29 |
| 202950_at | crystallin, zeta (quinone reductase) | CRYZ | 1p31-p22 | 0.0026 | 6.14 | 5.66 | -1.39 |
| 213629_x_at | metallothionein 1F | MT1F | 16q13 | 0.0028 | 9.93 | 9.49 | -1.36 |
| 223419_at | F-box and WD repeat domain containing 9 | FBXW9 | 19p13.13 | 0.0029 | 6.35 | 6.03 | -1.26 |
| 231319_x_at | kinesin family member 9 | KIF9 | 3p21.31 | 0.0029 | 7.40 | 7.06 | -1.27 |
| 1552921_a_at | fidgetin-like 1 | FIGNL1 | 7p12.2 | 0.0030 | 4.91 | 5.23 | 1.25 |
| 231728_at | calcyphosine | CAPS | 19p13.3 | 0.0030 | 5.83 | 5.33 | -1.41 |
| 209349_at | RAD50 homolog (S. cerevisiae) | RAD50 | 5q31 | 0.0032 | 5.19 | 5.57 | 1.30 |
| 231084_at | chromosome 10 open reading frame 79 | C10orf79 | 10q25.1 | 0.0033 | 5.81 | 5.14 | -1.59 |
| 222780_s_at | brain and acute leukemia, cytoplasmic | BAALC | 8q22.3 | 0.0033 | 8.21 | 8.64 | 1.35 |
| 219383_at | protor-2 | FLJ14213 | 11p13-p12 | 0.0033 | 3.99 | 4.33 | 1.26 |
| 240275_at | armadillo repeat containing 3 | ARMC3 | 10p12.31 | 0.0033 | 5.29 | 4.95 | -1.27 |
| 212793_at | dishevelled associated activator of morphogenesis 2 | DAAM2 | 6p21.2 | 0.0035 | 8.57 | 9.19 | 1.53 |
| 222028_at | zinc finger protein 45 | ZNF45 | 19q13.2 | 0.0036 | 4.90 | 5.22 | 1.25 |
| 220026_at | chloride channel, calcium activated, family member 4 | CLCA4 | 1p31-p22 | 0.0037 | 5.45 | 5.92 | 1.38 |
| 204472_at | GTP binding protein overexpressed in skeletal muscle | GEM | 8q13-q21 | 0.0037 | 6.42 | 5.93 | -1.40 |
| 222791_at | round spermatid basic protein 1 | RSBN1 | 1p13.2 | 0.0037 | 7.38 | 7.04 | -1.26 |
| 225014_at | hypothetical gene supported by BC032431 | LOC389203 | 4p15.2 | 0.0038 | 7.44 | 7.78 | 1.26 |
| 224566_at | trophoblast-derived noncoding RNA | TncRNA | 11q13.1 | 0.0038 | 7.36 | 8.14 | 1.72 |
| 225951_s_at | Chromodomain helicase DNA binding protein 2 | CHD2 | 15q26 | 0.0038 | 8.78 | 9.15 | 1.30 |
| 200632_s_at | N-myc downstream regulated gene 1 | NDRG1 | 8q24.3 | 0.0039 | 9.27 | 9.77 | 1.41 |
| 208651_x_at | CD24 molecule | CD24 | 6q21 | 0.0040 | 7.63 | 6.97 | -1.58 |
| 212450_at | KIAA0256 gene product | KIAA0256 | 15q21.1 | 0.0040 | 8.81 | 9.18 | 1.29 |
| 227401_at | interleukin 17D | IL17D | 13q12.11 | 0.0041 | 8.32 | 8.67 | 1.27 |
| 209841_s_at | leucine rich repeat neuronal 3 | LRRN3 | 7q31.1 | 0.0043 | 8.69 | 8.35 | -1.26 |
| 210163_at | chemokine (C-X-C motif) ligand 11 | CXCL11 | 4q21.2 | 0.0043 | 3.74 | 3.42 | -1.25 |
| 204221_x_at | GLI pathogenesis-related 1 | GLIPR1 | 12q21.2 | 0.0044 | 6.12 | 5.52 | -1.51 |
| 230204_at | Hyaluronan and proteoglycan link protein 1 | HAPLN1 | 5q14.3 | 0.0045 | 5.30 | 5.64 | 1.26 |
| 222439_s_at | thyroid hormone receptor associated protein 3 | THRAP3 | 1p34.3 | 0.0046 | 6.28 | 5.93 | -1.28 |
| 214369_s_at | RAS guanyl releasing protein 2 (calcium and DAG-regulated) | RASGRP2 | 11q13 | 0.0047 | 5.94 | 6.32 | 1.30 |
| 202514_at | discs, large homolog 1 (Drosophila) | DLG1 | 3q29 | 0.0047 | 7.77 | 8.21 | 1.36 |
| 210033_s_at | sperm associated antigen 6 | SPAG6 | 10p12.2 | 0.0049 | 5.03 | 4.53 | -1.42 |
| 208950_s_at | aldehyde dehydrogenase 7 family, member A1 | ALDH7A1 | 5q31 | 0.0050 | 7.19 | 7.85 | 1.58 |
| 238116_at | dynein, light chain, roadblock-type 2 | DYNLRB2 | 16q23.3 | 0.0050 | 5.71 | 4.92 | -1.72 |
| 218656_s_at | lipoma HMGIC fusion partner | LHFP | 13q12 | 0.0051 | 7.43 | 7.80 | 1.29 |
| 201185_at | HtrA serine peptidase 1 | HTRA1 | 10q26.3 | 0.0051 | 9.50 | 9.98 | 1.40 |
| 203464_s_at | epsin 2 | EPN2 | 17p11.2 | 0.0051 | 7.45 | 7.80 | 1.27 |
| 205695_at | serine dehydratase | SDS | 12q24.13 | 0.0051 | 6.21 | 6.79 | 1.49 |
| 210427_x_at | annexin A2 | ANXA2 | 15q21-q22 | 0.0052 | 8.15 | 7.76 | -1.31 |
| 202259_s_at | NEDD4 binding protein 2-like 2 | N4BP2L2 | 13q13.1 | 0.0053 | 6.35 | 6.86 | 1.43 |
| 216379_x_at | CD24 molecule | CD24 | 6q21 | 0.0053 | 8.63 | 7.58 | -2.08 |
| 209096_at | ubiquitin-conjugating enzyme E2 variant 2 | UBE2V2 | 8q11.21 | 0.0053 | 7.45 | 7.78 | 1.26 |
| 220591_s_at | EF-hand domain (C-terminal) containing 2 | EFHC2 | Xp11.3 | 0.0054 | 5.25 | 4.87 | -1.30 |
| 206483_at | leucine rich repeat containing 6 | LRRC6 | 8q24.22 | 0.0054 | 6.51 | 6.16 | -1.27 |
| 209072_at | myelin basic protein | MBP | 18q23 | 0.0054 | 12.84 | 13.37 | 1.44 |
| 218033_s_at | Stannin | SNN | 16p13 | 0.0056 | 7.03 | 7.48 | 1.37 |
| 213748_at | tripartite motif-containing 66 | TRIM66 | 11p15.4 | 0.0057 | 6.79 | 6.42 | -1.29 |
| 202270_at | guanylate binding protein 1, interferon-inducible, 67kDa | GBP1 | 1p22.2 | 0.0058 | 4.21 | 3.78 | -1.35 |
| 240065_at | family with sequence similarity 81, member B | FAM81B | 5q15 | 0.0059 | 5.11 | 4.51 | -1.53 |
| 202908_at | Wolfram syndrome 1 (wolframin) | WFS1 | 4p16 | 0.0059 | 8.85 | 8.39 | -1.37 |
| 204036_at | lysophosphatidic acid receptor 1 | LPAR1 | 9q31.3 | 0.0060 | 8.99 | 9.69 | 1.63 |
| 242214_at | ribosomal protein S27a /// ubiquitin B /// ubiquitin C | RPS27A /// UBB /// UBC | 12q24.3 /// 17p12-p11.2 /// 2p16 | 0.0061 | 7.67 | 8.01 | 1.26 |
| 229169_at | tetratricopeptide repeat domain 18 | TTC18 | 10q22.2 | 0.0062 | 4.73 | 4.41 | -1.25 |
| 221718_s_at | A kinase (PRKA) anchor protein 13 | AKAP13 | 15q24-q25 | 0.0062 | 7.59 | 7.23 | -1.29 |
| 232063_x_at | phenylalanyl-tRNA synthetase, beta subunit | FARSB | 2q36.1 | 0.0062 | 6.54 | 6.17 | -1.29 |
| 207323_s_at | myelin basic protein | MBP | 18q23 | 0.0063 | 11.90 | 12.56 | 1.59 |
| 212419_at | zinc finger, CCHC domain containing 24 | ZCCHC24 | 10q22.3 | 0.0063 | 8.88 | 9.33 | 1.36 |
| 221776_s_at | bromodomain containing 7 | BRD7 | 16q12 | 0.0064 | 6.80 | 7.19 | 1.31 |
| 229331_at | spermatogenesis associated 18 homolog (rat) | SPATA18 | 4q12 | 0.0065 | 4.30 | 3.97 | -1.26 |
| 218102_at | 2-deoxyribose-5-phosphate aldolase homolog (C. elegans) | DERA | 12p12.3 | 0.0065 | 5.49 | 5.81 | 1.25 |
| 209771_x_at | CD24 molecule | CD24 | 6q21 | 0.0066 | 8.71 | 7.67 | -2.06 |
| 221654_s_at | ubiquitin specific peptidase 3 | USP3 | 15q22.3 | 0.0066 | 5.12 | 5.51 | 1.32 |
| 217597_x_at | RAB40B, member RAS oncogene family | RAB40B | 17q25.3 | 0.0066 | 7.02 | 7.36 | 1.27 |
| 224463_s_at | chromosome 11 open reading frame 70 | C11orf70 | 11q22.1 | 0.0068 | 5.47 | 4.90 | -1.49 |
| 208650_s_at | CD24 molecule | CD24 | 6q21 | 0.0069 | 5.23 | 4.34 | -1.84 |
| 1555725_a_at | regulator of G-protein signaling 5 | RGS5 | 1q23.1 | 0.0069 | 8.64 | 9.10 | 1.37 |
| 228376_at | glycoprotein, alpha-galactosyltransferase 1 | GGTA1 | 9q33.2-q34.11 | 0.0071 | 7.51 | 7.95 | 1.36 |
| 221748_s_at | tensin 1 | TNS1 | 2q35-q36 | 0.0071 | 7.14 | 7.63 | 1.40 |
| 217165_x_at | metallothionein 1F | MT1F | 16q13 | 0.0074 | 9.93 | 9.59 | -1.26 |
| 213225_at | protein phosphatase 1B (formerly 2C), magnesium-dependent, beta isoform | PPM1B | 2p21 | 0.0074 | 8.37 | 8.72 | 1.28 |
| 212899_at | cell division cycle 2-like 6 (CDK8-like) | CDC2L6 | 6q21 | 0.0075 | 8.00 | 8.44 | 1.35 |
| 209686_at | S100 calcium binding protein B | S100B | 21q22.3 | 0.0075 | 9.37 | 9.85 | 1.39 |
| 202125_s_at | trafficking protein, kinesin binding 2 | TRAK2 | 2q33 | 0.0075 | 8.39 | 8.73 | 1.26 |
| 213164_at | solute carrier family 5 (sodium/myo-inositol cotransporter), member 3 | SLC5A3 | 21q22.12 | 0.0076 | 9.17 | 8.67 | -1.42 |
| 229902_at | fms-related tyrosine kinase 4 | FLT4 | 5q35.3 | 0.0076 | 4.21 | 4.56 | 1.28 |
| 209504_s_at | pleckstrin homology domain containing, family B (evectins) member 1 | PLEKHB1 | 11q13.5-q14.1 | 0.0076 | 10.42 | 10.90 | 1.39 |
| 205474_at | cytokine receptor-like factor 3 | CRLF3 | 17q11.2 | 0.0078 | 7.61 | 7.22 | -1.30 |
| 203801_at | mitochondrial ribosomal protein S14 | MRPS14 | 1q23-q25 | 0.0080 | 6.25 | 6.59 | 1.27 |
| 201929_s_at | plakophilin 4 | PKP4 | 2q23-q31 | 0.0082 | 8.67 | 9.25 | 1.50 |
| 209000_s_at | septin 8 | 8-Sep | 5q31 | 0.0082 | 7.98 | 8.78 | 1.75 |
| 204850_s_at | doublecortin | DCX | Xq22.3|Xq22.3-q23 | 0.0084 | 6.37 | 6.71 | 1.26 |
| 222410_s_at | sorting nexin 6 | SNX6 | 14q13.2 | 0.0086 | 8.39 | 8.76 | 1.29 |
| 214783_s_at | annexin A11 | ANXA11 | 10q23 | 0.0086 | 6.70 | 6.30 | -1.32 |
| 213622_at | collagen, type IX, alpha 2 | COL9A2 | 1p33-p32 | 0.0087 | 6.37 | 6.77 | 1.32 |
| 231044_at | chromosome 1 open reading frame 194 | C1orf194 | 1p13.3 | 0.0089 | 7.75 | 7.35 | -1.32 |
| 201337_s_at | vesicle-associated membrane protein 3 (cellubrevin) | VAMP3 | 1p36.23 | 0.0089 | 6.41 | 6.94 | 1.45 |
| 219728_at | myotilin | MYOT | 5q31 | 0.0089 | 5.53 | 6.13 | 1.51 |
| 227632_at | TBC1 domain family, member 24 | TBC1D24 | 16p13.3 | 0.0089 | 6.59 | 6.17 | -1.34 |
| 220230_s_at | cytochrome b5 reductase 2 | CYB5R2 | 11p15.4 | 0.0089 | 6.28 | 6.87 | 1.50 |
| 223058_at | family with sequence similarity 107, member B | FAM107B | 10p13 | 0.0089 | 7.82 | 8.61 | 1.72 |
| 230493_at | shisa homolog 2 (Xenopus laevis) | SHISA2 | 13q12.13 | 0.0089 | 6.22 | 6.84 | 1.54 |
| 216988_s_at | protein tyrosine phosphatase type IVA, member 2 | PTP4A2 | 1p35 | 0.0090 | 9.57 | 9.97 | 1.33 |
| 1569003_at | transmembrane protein 49 | TMEM49 | 17q23.1 | 0.0091 | 6.54 | 6.14 | -1.33 |
| 210657_s_at | septin 4 | 4-Sep | 17q22-q23 | 0.0092 | 8.75 | 9.48 | 1.65 |
| 200920_s_at | B-cell translocation gene 1, anti-proliferative | BTG1 | 12q22 | 0.0092 | 8.31 | 7.98 | -1.26 |
| 225473_at | chromosome 20 open reading frame 117 | C20orf117 | 20q11.23 | 0.0092 | 6.47 | 6.83 | 1.28 |
| 210240_s_at | cyclin-dependent kinase inhibitor 2D (p19, inhibits CDK4) | CDKN2D | 19p13 | 0.0092 | 7.71 | 8.04 | 1.25 |
| 227064_at | ankyrin repeat domain 40 | ANKRD40 | 17q21.33 | 0.0095 | 9.22 | 9.57 | 1.28 |
| 213998_s_at | DEAD (Asp-Glu-Ala-Asp) box polypeptide 17 | DDX17 | 22q13.1 | 0.0099 | 6.58 | 5.98 | -1.52 |
| 237974_at | abhydrolase domain containing 12B | ABHD12B | 14q22.1 | 0.0099 | 4.78 | 5.30 | 1.43 |
| 211994_at | WNK lysine deficient protein kinase 1 | WNK1 | 12p13.3 | 0.0100 | 9.69 | 10.02 | 1.25 |
