# Supplementary material for: Gene Expression Changes in the Prefrontal Cortex, Anterior Cingulate Cortex and Nucleus Accumbens of Mood Disorders Subjects That Committed Suicide
Source: PLoS One. 2012 Apr 30;7(4):e35367. doi: 10.1371/journal.pone.0035367 (PMC3340369; doi:10.1371/journal.pone.0035367)
Supplement: Table S6 — Accession numbers for the chips included in the study. (DOC) [file pone.0035367.s006.doc]

**Supporting Table 6.** Accession numbers for the chips included in the study.

| **Accession** | **Title** | **Sample Type** | **Taxonomy** | **Channels** | **Platform** | **Series** |
| --- | --- | --- | --- | --- | --- | --- |
| **GSM145064** | I_U133Plus_73_AnCg | RNA | Homo sapiens | 1 | GPL4557 | GSE6306 |
| **GSM145076** | I_U133Plus_94_AnCg | RNA | Homo sapiens | 1 | GPL4557 | GSE6306 |
| **GSM145085** | I_U133Plus_108_AnCg | RNA | Homo sapiens | 1 | GPL4557 | GSE6306 |
| **GSM145087** | I_U133Plus_110_AnCg | RNA | Homo sapiens | 1 | GPL4557 | GSE6306 |
| **GSM145090** | I_U133Plus_113_AnCg | RNA | Homo sapiens | 1 | GPL4557 | GSE6306 |
| **GSM145092** | I_U133Plus_115_AnCg | RNA | Homo sapiens | 1 | GPL4557 | GSE6306 |
| **GSM145093** | I_U133Plus_116_AnCg | RNA | Homo sapiens | 1 | GPL4557 | GSE6306 |
| **GSM145095** | I_U133Plus_119_AnCg | RNA | Homo sapiens | 1 | GPL4557 | GSE6306 |
| **GSM145096** | I_U133Plus_120_AnCg | RNA | Homo sapiens | 1 | GPL4557 | GSE6306 |
| **GSM145097** | I_U133Plus_121_AnCg | RNA | Homo sapiens | 1 | GPL4557 | GSE6306 |
| **GSM145098** | I_U133Plus_122_AnCg | RNA | Homo sapiens | 1 | GPL4557 | GSE6306 |
| **GSM145099** | I_U133Plus_123_AnCg | RNA | Homo sapiens | 1 | GPL4557 | GSE6306 |
| **GSM145100** | I_U133Plus_124_AnCg | RNA | Homo sapiens | 1 | GPL4557 | GSE6306 |
| **GSM145101** | I_U133Plus_125_AnCg | RNA | Homo sapiens | 1 | GPL4557 | GSE6306 |
| **GSM145102** | I_U133Plus_126_AnCg | RNA | Homo sapiens | 1 | GPL4557 | GSE6306 |
| **GSM145410** | D_U133Plus_26_DLPFC | RNA | Homo sapiens | 1 | GPL4557 | GSE6306 |
| **GSM145419** | D_U133Plus_33_DLPFC | RNA | Homo sapiens | 1 | GPL4557 | GSE6306 |
| **GSM145422** | D_U133Plus_34_DLPFC | RNA | Homo sapiens | 1 | GPL4557 | GSE6306 |
| **GSM145425** | D_U133Plus_36_DLPFC | RNA | Homo sapiens | 1 | GPL4557 | GSE6306 |
| **GSM145428** | D_U133Plus_37_DLPFC | RNA | Homo sapiens | 1 | GPL4557 | GSE6306 |
| **GSM145430** | D_U133Plus_38_DLPFC | RNA | Homo sapiens | 1 | GPL4557 | GSE6306 |
| **GSM145451** | D_U133Plus_59_DLPFC | RNA | Homo sapiens | 1 | GPL4557 | GSE6306 |
| **GSM145465** | D_U133Plus_73_DLPFC | RNA | Homo sapiens | 1 | GPL4557 | GSE6306 |
| **GSM145486** | D_U133Plus_94_DLPFC | RNA | Homo sapiens | 1 | GPL4557 | GSE6306 |
| **GSM145501** | D_U133Plus_110_DLPFC | RNA | Homo sapiens | 1 | GPL4557 | GSE6306 |
| **GSM145504** | D_U133Plus_113_DLPFC | RNA | Homo sapiens | 1 | GPL4557 | GSE6306 |
| **GSM145506** | D_U133Plus_115_DLPFC | RNA | Homo sapiens | 1 | GPL4557 | GSE6306 |
| **GSM145507** | D_U133Plus_116_DLPFC | RNA | Homo sapiens | 1 | GPL4557 | GSE6306 |
| **GSM145510** | D_U133Plus_119_DLPFC | RNA | Homo sapiens | 1 | GPL4557 | GSE6306 |
| **GSM145511** | D_U133Plus_120_DLPFC | RNA | Homo sapiens | 1 | GPL4557 | GSE6306 |
| **GSM145512** | D_U133Plus_121_DLPFC | RNA | Homo sapiens | 1 | GPL4557 | GSE6306 |
| **GSM145513** | D_U133Plus_122_DLPFC | RNA | Homo sapiens | 1 | GPL4557 | GSE6306 |
| **GSM145514** | D_U133Plus_123_DLPFC | RNA | Homo sapiens | 1 | GPL4557 | GSE6306 |
| **GSM145515** | D_U133Plus_124_DLPFC | RNA | Homo sapiens | 1 | GPL4557 | GSE6306 |
| **GSM145516** | D_U133Plus_125_DLPFC | RNA | Homo sapiens | 1 | GPL4557 | GSE6306 |
| **GSM145517** | D_U133Plus_126_DLPFC | RNA | Homo sapiens | 1 | GPL4557 | GSE6306 |
| **GSM146059** | M_U133Plus_106_nAcc | RNA | Homo sapiens | 1 | GPL4557 | GSE6306 |
| **GSM146061** | M_U133Plus_107_nAcc | RNA | Homo sapiens | 1 | GPL4557 | GSE6306 |
| **GSM146062** | M_U133Plus_108_nAcc | RNA | Homo sapiens | 1 | GPL4557 | GSE6306 |
| **GSM146064** | M_U133Plus_109_nAcc | RNA | Homo sapiens | 1 | GPL4557 | GSE6306 |
| **GSM146065** | M_U133Plus_110_nAcc | RNA | Homo sapiens | 1 | GPL4557 | GSE6306 |
| **GSM146067** | M_U133Plus_111_nAcc | RNA | Homo sapiens | 1 | GPL4557 | GSE6306 |
| **GSM146069** | M_U133Plus_112_nAcc | RNA | Homo sapiens | 1 | GPL4557 | GSE6306 |
| **GSM146070** | M_U133Plus_113_nAcc | RNA | Homo sapiens | 1 | GPL4557 | GSE6306 |
| **GSM146072** | M_U133Plus_114_nAcc | RNA | Homo sapiens | 1 | GPL4557 | GSE6306 |
| **GSM146073** | M_U133Plus_115_nAcc | RNA | Homo sapiens | 1 | GPL4557 | GSE6306 |
| **GSM146074** | M_U133Plus_116_nAcc | RNA | Homo sapiens | 1 | GPL4557 | GSE6306 |
| **GSM146076** | M_U133Plus_117_nAcc | RNA | Homo sapiens | 1 | GPL4557 | GSE6306 |
| **GSM146078** | M_U133Plus_118_nAcc | RNA | Homo sapiens | 1 | GPL4557 | GSE6306 |
| **GSM146079** | M_U133Plus_119_nAcc | RNA | Homo sapiens | 1 | GPL4557 | GSE6306 |
| **GSM146080** | M_U133Plus_120_nAcc | RNA | Homo sapiens | 1 | GPL4557 | GSE6306 |
| **GSM146081** | M_U133Plus_121_nAcc | RNA | Homo sapiens | 1 | GPL4557 | GSE6306 |
| **GSM146082** | M_U133Plus_122_nAcc | RNA | Homo sapiens | 1 | GPL4557 | GSE6306 |
| **GSM146083** | M_U133Plus_123_nAcc | RNA | Homo sapiens | 1 | GPL4557 | GSE6306 |
| **GSM146084** | M_U133Plus_124_nAcc | RNA | Homo sapiens | 1 | GPL4557 | GSE6306 |
| **GSM146085** | M_U133Plus_125_nAcc | RNA | Homo sapiens | 1 | GPL4557 | GSE6306 |
| **GSM146086** | M_U133Plus_126_nAcc | RNA | Homo sapiens | 1 | GPL4557 | GSE6306 |
